# Multifunctional Bilayer Wound Dressing Composed of Immediate Release Layer of Ofloxacin and Sustained Release Layer of Bergamot Oil

**DOI:** 10.3390/pharmaceutics17121589

**Published:** 2025-12-10

**Authors:** Mehar Un Nisa, Ikram Ullah Khan, Yousaf Kamal, Usra, Zunera Chaudhary, Ghulam Hussain, Muhammad Irfan, Syed Haroon Khalid, Sajid Asghar, Hafeez Ullah Khan, Safirah Maheen, Syed Adnan Ali Shah, Thierry F. Vandamme

**Affiliations:** 1 Department of Pharmaceutics, Faculty of Pharmaceutical Sciences, Government College University Faisalabad, Faisalabad 38000, Pakistan; mehar.2172@gmail.com (M.U.N.); manipharma1@gmail.com (M.I.); haroonkhalid80@gmail.com (S.H.K.); sajuhappa@gmail.com (S.A.); 2 Hamdard Institute of Pharmaceutical Sciences, Hamdard University Karachi, Islamabad Campus, Islamabad 45550, Pakistan; yousafpharmacist1@gmail.com; 3 Department of Physiology, Faculty of Life Sciences, Government College University Faisalabad, Faisalabad 38000, Pakistan; usrakhan1990@gmail.com (U.); hussain806@gmail.com (G.H.); 4 Department of Pharmacology, Faculty of Pharmaceutical Sciences, Government College University Faisalabad, Faisalabad 38000, Pakistan; zunerach@yahoo.com; 5 Department of Pharmaceutics, Faculty of Pharmacy, Universiti Teknologi Mara, Bandar Puncak Alam 42300, Selangor, Malaysia; 6 Department of Pharmaceutics, College of Pharmacy, University of Sargodha, Sargodha 40100, Pakistan; hafeezullah.khan@uos.edu.pk (H.U.K.); msafirah@yahoo.com (S.M.); 7 Faculty of Pharmacy, Universiti Teknologi MARA Cawangan Selangor Kampus Puncak Alam, Bandar Puncak Alam 42300, Selangor, Malaysia; syedadnan@uitm.edu.my; 8 Atta-ur-Rahman Institute for Natural Product Discovery (AuRIns), Universiti Teknologi MARA Cawangan Selangor Kampus Puncak Alam, Bandar Puncak Alam 42300, Selangor, Malaysia; 9 Centre de Recherche en Biomédecine de Strasbourg (CRBS), Inserm/Unistra, UMR 1260 Regenerative NanoMedecine, Université de Strasbourg, 1 Rue Eugène Boeckel, 67000 Strasbourg, France; vandamme@unistra.fr

**Keywords:** bilayer films, hydrogel, bergamot oil, ofloxacin, wound healing

## Abstract

**Background**: Wound healing is a typical biological process that the human body accomplishes through well-defined stages. The complexity of the healing process continues to be a significant health challenge. Multifunctional polymeric bilayer wound dressings have emerged as a new treatment option, as they resemble the bilayer structure of skin. **Methods**: Here, we developed a bilayer film with two distinct features, i.e., a primary sodium alginate (Na-Alg)-based sustained release layer incorporated with bergamot essential oil (BEO) and a secondary immediate release layer of hydroxypropyl methyl cellulose (HPMC) and hydroxyethyl cellulose (HEC) loaded with the antibacterial drug ofloxacin (OFX). Using the double solvent casting technique. **Results**: The resultant bilayer films exhibited good folding endurance and swelling capability. The antibacterial potential was appraised by assessing their capability to hinder the growth of *S. aureus* (40 mm zone of inhibition) and *E. coli* (46 mm zone of inhibition). A DPPH assay confirmed the anti-oxidant ability of the incorporated essential oil. The outcomes of the X-ray diffraction and FTIR analysis support the even and complete dispersion of the oil and drug into the polymeric matrix without any unwanted interaction. The SEM results revealed a slightly microstructured surface view, while microporous structures were discovered in the cross-section due to the presence of the oil and drug. In the in vivo wound model, the developed bilayer films demonstrated a quicker rate of wound closure (98.5% in 12 days) and avoided wound infection. Histological studies verified that the created dressing enhanced the deposition of mature collagen and promoted epithelialization. **Conclusions**: As a result, the unique blend of anti-inflammatory and antibacterial properties in bilayer films can significantly offer fresh perspectives for developing sophisticated, multipurpose wound dressings to hasten the healing of cutaneous wounds.

## 1. Introduction

The maintenance of physiological homeostasis in the human body is reliant upon the integrity of healthy skin. The skin, the largest organ in the human body, is sometimes damaged during surgery due to accidental injury or any other trauma, which could therefore be lethal [1]. The body replaces damaged tissues with healthy ones through a well-defined process known as wound healing. Restoring the integrity of damaged tissue is a dynamic and complex process that involves several cellular and matrix components working together. Rapid wound closure and leaving minimal scars are the main objectives of wound care. In order to create the ideal healing environment for wound healing, wound management is crucial [2]. Unfortunately, a variety of problems may occur and affect the time it takes to heal from injuries. For instance, bacteria that readily colonize wounds, such as *Escherichia coli* and *Staphylococcus aureus*, can result in complicated and severe infections [3].

Wounds were traditionally covered with bandages, gauze, lint, cotton, and wool. Because of their adhesion to the site, low capacity to absorb fluid exudates, permeability to germs, and inadequate wound coverage, these dry dressings may cause further wound damage [4]. Depending on the extent of the wound, the ideal wound dressing may provide moisture and occlusion to protect against infections and contamination, facilitate debridement, and permit easy application and removal to prevent trauma associated with dressings [5]. Drug-loaded wound dressings are occasionally used to treat wounds locally, such as to prevent subsequent infections or to manage pain, particularly in chronic wounds. The market for wound care management offers a wide range of wound care solutions that are intended to treat both acute and chronic wounds [6].

Advanced dressings can supply bioactive substances like growth factors, antioxidants, anti-inflammatory constituents, and antibiotics that aid in the healing process. Hydrogel films are a promising class of materials that have already entered the commercial market and have demonstrated successful performance. Hydrogel films are superior to traditional synthetic wound dressings because they may expand without polymer degradation, giving them properties that are similar to those of soft tissue. Hydrogel dressings keep wounds moist, creating a physical barrier that prevents infection and microbial penetration while promoting keratinocyte migration and fibroblast development [7].

Prior research has revealed that, in comparison to single-component films, composite films offer greater mechanical, transferable, and physical characteristics [8]. Bilayer composite film systems, based on various biopolymers, have garnered a lot of interest lately in the domains of food technology, medicine, and bioengineering due to their superior mechanical properties, ease of preparation compared to other film formulations, and superior moisture retention capabilities. Bilayer films have a heterogeneous structure, with the intrinsic properties of each polymer being preserved on the corresponding layer, unlike blends [9]. Bilayer hydrogel dressings for the treatment of skin conditions are given special attention in novel wound therapies. The previously mentioned drawbacks of conventional wound dressings have led to the development of several bilayer dressings that imitate the top and bottom layers of the epidermis. A thick top layer and a thin porous biodegradable base layer make up the bilayer sheets [10].

A critical first step in making a good wound dressing is selecting the appropriate materials for the synthesis of the top layer and sublayers. Sodium alginate (Na-Alg), one of the naturally occurring linear polysaccharides, is extracted from brown algae (Phaeophyceae). Its remarkable properties include high hygroscopicity, detachability, oxygen permeability, cell adhesion, and biocompatibility, which have led to its widespread application in wound dressings. Na-Alg-based hydrogels can also promote the growth of epidermal cell proliferation [11]. In order to create a thin matrix with rapid disintegration and favorable mechanical properties, water-soluble polymers, which might be natural, semisynthetic, or synthetic, are usually used in the production of drug-loaded fast-dissolving films. Commonly utilized hydrophilic cellulose derivatives include hydroxyethyl cellulose (HEC), hydroxypropyl cellulose (HPC), hydroxypropyl methylcellulose (HPMC), polyvinyl alcohol (PVA), and polyvinylpyrrolidone (PVP) [12]. Phenols, alkaloids, flavonoids, tannins, terpenoids, fatty acids, and essential oils are examples of bioactive secondary metabolites from plants that can be employed to treat wounds. These active ingredients have antibacterial, antifungal, antioxidant, and anti-inflammatory attributes that may accelerate the healing process by affecting one of the stages of recovery [13]. Significant evidence supports EO’s antibacterial, anti-inflammatory, and antioxidant qualities—three essential components in the treatment of chronic wounds [14]. The precursor molecules that exacerbate the inflammatory process—nitrogen species, proinflammatory cytokines, NF-κ B, and reactive oxygen species (ROS)—are reduced by EOs. It was discovered that bergamot essential oil (EO) significantly lowers levels of inflammatory markers, such as tumor necrosis factor (TNF-α), vascular endothelial growth factor (VEGF), and IL-6 [15]. The present work aimed to develop a multifunctional bilayer wound dressing with antibacterial, anti-inflammatory, and antioxidant properties by utilizing natural and synthetic polymers that encapsulate essential oil alongside antibacterial medication. We proposed a Na-Alg-HPMC/HEC-based bilayer film with the goal of investigating its potential for the slow release of oil and as an immediate-release antibacterial agent for the management of wound healing.

## 2. Materials and Methods

### 2.1. Materials

Sodium alginate (Na-Alg) was purchased from Daejung Chemicals, Siheung-si, Republic of Korea, HPMC E5 was gifted by Saffron Pharmaceuticals (Faisalabad, Pakistan), HEC was obtained from Sigma-Aldrich (St. Louis, MO, USA), calcium chloride and propylene glycol were obtained from Sigma-Aldrich, Tween 80^®^ was obtained from Daejung Chemicals (Republic of Korea), ofloxacin (OFX) was gifted by Saffron Pharmaceuticals, bergamot essential oil (BEO) and frankincense essential oil (FEO) were purchased from Chiltan Pure (Lahore, Pakistan), chamomile essential oil (CEO) was purchased from Sukoon by Nutrify (Faisalabad, Pakistan), and nutrient agar was obtained from Oxoid Ltd. (Basingstoke, UK). All chemicals employed in formulation were of analytical grade.

### 2.2. Selection and Optimization of Formulation

#### 2.2.1. Optimization of Primary Layer

Number of trials were conducted on single-layer films separately for the selection of essential oil and optimization of oil and drug concentration to be used in final bilayer formulation. Primary layer formulations were prepared with three different oils at different concentrations, with fixed concentrations of other excipients. Formulations of primary layer are mentioned in Table 1.

#### 2.2.2. Optimization of Secondary Layer

Similarly, secondary layer was also formulated with three different concentrations of drug, and resultant formulations are listed in Table 2. All the single layers were evaluated for pre-formulation studies.

### 2.3. Synthesis of Bilayer Film

Bilayer film was prepared by double solvent casting method with slight modification, as previously reported [17]. Optimized B2 and D2 layers were used in bilayer film development.

#### 2.3.1. Preparation of Primary Layer

To create the top layer of the bilayer film, sodium alginate 2% *w*/*v* was dissolved in distilled water at 60–70 °C while being continuously stirred (700 rpm) for one hr. To stabilize the essential oil, Tween 80 (4% *w*/*w* of oil) as a surfactant and propylene glycol (20% *w*/*w* of polymer) as a plasticizer were added to the polymeric solution and thoroughly mixed. Afterwards, essential oil (25% *w*/*w* of polymer) was added to this polymeric solution. Using a bath sonicator, air bubbles in solution were eliminated. The resultant solution was cast in a glass Petri plate of about 90 mm in diameter. After 30 min, a freshly made 4–5 mL calcium chloride (0.5% *w*/*v*) crosslinking solution was evenly sprayed over the cast film solution using a spray bottle. This layer was left to dry at room temperature.

#### 2.3.2. Preparation of Secondary Layer

**Figure 1:**
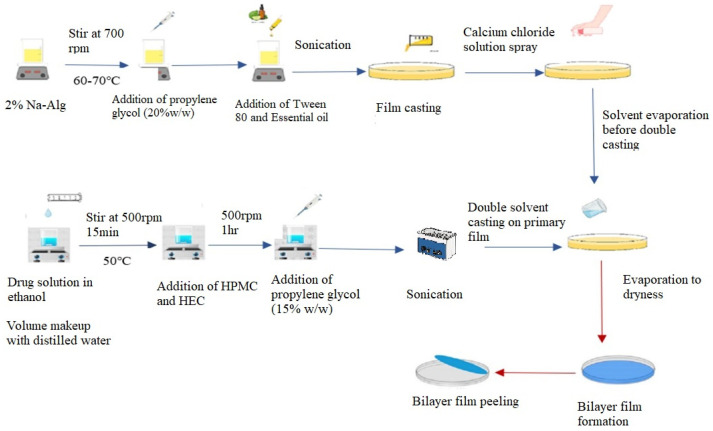
Graphical representation of double solvent casting technique for synthesis of bilayer film.

After the drying of the first layer, the second layer was cast. In order to attain this, OFX (5% *w*/*w* of polymer) was dissolved in 4 mL of ethanol while being constantly stirred without heat. After the drug was thoroughly dissolved, the volume of the solution was increased to 25 mL using distilled water along with the stepwise addition of HPMC (2.5% *w*/*v*) and HEC (0.5% *w*/*v*) while stirring continuously (700 rpm) at 50–60 °C until a clear solution was formed. Afterwards, propylene glycol (15 percent *w*/*w* of polymer) was added to this solution. The solution’s air bubbles were eliminated using a sonicator, and the polymeric solution of secondary layer was cast onto previously dried primary film and allowed to air dry (Figure 1). The resultant bilayer film was taken out of the Petri plate, cut to the proper size of 1.5 × 1.5 cm^2^, wrapped in aluminum foil, and stored until further use.

#### 2.3.3. Formulation of Bilayer Film

By using optimized oil and drug concentration in both sustained and immediate layers (Table 3), respectively, final bilayer formulations were prepared with predetermined concentrations of polymers, as were used in the single layers.

### 2.4. Preformulation Studies

#### 2.4.1. Physical Examination

Oil- and drug-loaded films were evaluated for color and physical appearance [18].

#### 2.4.2. Thickness and Weight Variation

The thickness of the film patches was measured using a handheld digital micrometer. Film patch (1.5 × 1.5 cm^2^) of each film formulation was subjected to arbitrary measurements, and the average findings were utilized to determine the film thickness (*n* = 10) [19]. On the other hand, the purpose of the weight variation test was to verify that the concentrations of the drug and essential oil were uniform in each film patch, without a significant deviation. This test ensures that the dosage for each film is precise. Each film patch’s (1.5 × 1.5 cm^2^) weight was measured using the OHAUS analytical weighing scale. The average weight and standard deviation were calculated for each film patch (*n* = 10) [17].

#### 2.4.3. Folding Endurance

The folding endurance test is used to assess the mechanical strength and flexibility of films. Ten randomly selected film patches were evaluated for each formulation. The film was bent up and down repeatedly at the same point until it broke or tore; the folding endurance value is the number of twists the film could withstand before breaking (*n* = 10) [20].

#### 2.4.4. Disintegration Time

Depending on the formulation, the disintegration time varies with the content of the film. Using the Petri dish approach, the disintegration time of drug-loaded immediate release films with varying drug concentrations and bilayer films was determined. The film was cut into a precisely measured 1.5 × 1.5 cm^2^ strip and put inside a Petri plate that had previously been filled with 10 mL of phosphate buffer saline (pH 7.4). A precise record of the disintegration time was made. Ten repetitions of this test were conducted [21].

#### 2.4.5. Swelling Studies

The swelling properties of the oil-loaded films were evaluated based on the degree of film hydration. To observe the swelling behavior of the hydrogel films, the pre-weighed films (1.5 × 1.5 cm^2^) were immersed in 10 mL of USP phosphate buffer solution (pH 7.4). At certain intervals (0.25, 0.5, 1, 2, 3, 4, 5, 6, 7, 8, 10, 12, and 24 hrs), films were removed from the buffer solution and weighed. The following formula was used to calculate the swelling index [22].

(1) $$Swelling\;index\left(SI\right)=\frac{\left(Wt-Wi\right)}{Wi}\times 100$$

where “Wt” stands for the final weight of the films at predetermined intervals, and “Wi” for the dried films’ initial weight.

### 2.5. Antibacterial Activity

The antibacterial activity of hydrogel films loaded with oil and drug against Gram-positive (*Staphylococcus aureus*) and Gram-negative (*Escherichia coli*) bacteria was evaluated using the agar disc diffusion method. A disc of a specific area of 8 mm from drug- and oil-loaded films (with three different concentrations) was placed over freshly grown bacterial colonies in solidified agar. Following a 24 h incubation period at 37 °C, the zone of inhibition was assessed (*n* = 3) [23].

### 2.6. Antioxidant Activity

The antioxidant activity of hydrogel films and pure oils was evaluated using the 2,2-diphenyl-1-picrylhydrazyl (DPPH) test, with a slight modification of the previously described 96-well plate method [24]. A total of 100 μL of 0.1M DPPH was combined with 125 μL of pure oil in the same concentrations as in the hydrogel films. Furthermore, to ensure proper extraction of the encapsulated content, appropriately weighed samples (25 mg) of produced films were soaked in roughly 5 mL of ethanol with continuous agitation for 48 h. To perform the DPPH technique, 100 μL of the film solution was mixed with 100 μL of 0.1 M DPPH solution. Following a mild shake with well plate shaker, the obtained mixture was placed in a darkened room for a complete hour. The absorbance was measured at 517 nm using ELIZA microplate reader. The results were recorded as the average ± standard deviation of the percentage of radical scavenging activity from three repeated experiments [25].

(2) $$\%\;scavenging\;activity=\frac{{Abs}_{blank\;}-{Abs}_{sample}}{{Abs}_{blank}}\times 100$$

### 2.7. Solid State Characterization

#### 2.7.1. Fourier Transform Infrared Spectroscopy (FTIR)

FTIR was utilized to identify the drug’s functional groups and the chemical interactions with other film excipients. For optimized, drug-loaded, oil-loaded, and bilayer film formulation, FTIR spectra were acquired. Analysis was conducted using Bruker FTIR, USA which has a scanning range of 4000–400 cm^−1^ [26].

#### 2.7.2. Scanning Electron Microscopy

To determine the sample’s cross-sectional view and surface morphology, scanning electron microscopy was utilized. FEI Quanta 450 FEG with EDX Detector, Oxford Instruments, X-Max 50 Scanning Electron Microscopy, Abingdon, UK was used to observe bi-layered films. The pure OFX, blank, and drug- and oil-loaded bilayer films’ surface morphology was assessed after gold coating [27].

#### 2.7.3. X-Ray Diffraction

XRD was utilized for a qualitative analysis of the crystallinity of OFX and hydrogel bilayer films. All the samples were examined using an X-ray diffractometer (RIGAKU ULTIMA IV HE-200IQ Tokyo, Japan) equipped with a CuKα radiation source running at 30 mA and 30 kV. At a scanning rate of 4°/min, all data was recorded at a 2θ diffraction angle between 5° and 40° [28].

#### 2.7.4. Thermogravimetry Analysis

The investigations were conducted using a PERKIN ELMER/STA 6000 thermogravimetric analyzer, USA. The test was conducted under nitrogen gas at temperatures between 25 to 400 °C at a rate of 20 °C/min. The bilayer film sample was heated in the sample pan. The weight loss was calculated using a temperature-versus-weight-loss % TGA curve [29].

#### 2.7.5. Differential Scanning Calorimetry

Thermal evaluation of pure drug, blank, and oil-loaded bilayer films was carried out using a PERKIN ELMER STA/600 series thermal analyzer, USA from 25 to 400 °C at a rate of 20 °C/min in the presence of a nitrogen stream [30].

### 2.8. Drug and Oil Content

Drug-loaded (immediate release) film with a specific area of 1.5 × 1.5 cm^2^ was placed in 20 mL of USP phosphate buffer with a pH of 7.4, and oil-loaded film was placed in a solution of PBS 7.4 and ethanol (60:40) for 24 h. The solution was then analyzed at 294 nm for OFX and 250 nm for bergamot oil using a UV-Visible spectrophotometer (CECIL Instruments, Milton, England). Previously constructed calibration curves were used to compute the drug entrapment efficiency and loading in triplicate [31].

(3) $$\%\;age\;entrapment\;efficiency=\frac{Actual\;drug\;or\;oil\;amount}{Theoratical\;drug\;or\;oil\;amount}\times 100$$

(4) $$Drug\;loading\left(\%\frac{w}{w}\right)=\frac{Amount\;of\;drug\;or\;oil\;in\;sample}{Weight\;of\;sample}\times 100$$

### 2.9. In Vitro Release

In vitro drug and oil release tests were performed in a Franz diffusion cell (LAB enterprise, Nasik, India) with phosphate buffer at pH 7.4 for drug release and phosphate buffer at pH 7.4 and ethanol ratio (60:40) for oil release, respectively, using a cellulose acetate artificial membrane with a specific dimension of 25 mm. The testing films were cut into 15 mm circles and placed on receptor compartments supported by cellulose acetate membranes. To prevent the medium from evaporating, the cell cap and sampling arms were sealed with paraffin film. Every experiment was conducted at 100 rpm and 32 °C. At predefined intervals, 0.5 mL of samples was removed and replaced with the same volume of freshly prepared pH 7.4 buffer. Samples were tested for bergamot oil at 250 nm and for ofloxacin at 294 nm using a UV-Visible spectrophotometer. The experiment was carried out precisely three times [32].

(5) $$\%\;age\;release=\frac{drug\;or\;oil\;concentration\;released}{actual\;drug\;or\;oil\;concentration}\times 100$$

The zero-order, first-order, Higuchi, and Korsmeyer–Peppas models were used to analyze release data following release studies in order to explicate the mechanism underlying drug release.

### 2.10. In Vivo Assays

#### 2.10.1. Wound Healing Activity

Wound healing study was conducted in rats using excision wound model, and all protocols were approved by the institutional Ethical Review Committee (ERC) of Government College University Faisalabad, Faisalabad, Pakistan (Ref. No: GCUF/ERC/423). In this study, Sprague Dawley rats weighing between 150 and 200 g were employed. Rats were acclimatized for one week in animal house facility at a relative humidity of 55 ± 5%, temperature of 25 ± 2 °C, and 12 h light/dark cycle using standard rodent diet and water ad libitum.

As indicated in Table 4, six groups of six healthy rats each were created by employing permuted block randomization. To render the rat unconscious, the animals were given an intraperitoneal injection of ketamine (0.1 g per Kg) and xylazine (0.01 g per kg) concoction to induce anesthesia. After shaving the specified area, it was cleaned with 70% ethanol. Using a biopsy punch, two full-thickness wounds of 4 mm in diameter were made on the rat’s dorsal side. After applying bilayer film specified for each group, the area was covered with elastic tape. These films and elastic bandages were changed every day. These wounds were then assessed by photographing each group of rats on days 1, 3, 5, 7, 10, and 12. The percentage of wound closure was measured with a digital vernier caliper, and the findings were calculated using a formula [33].

(6) $$\%\;wound\;contraction=Ao-\frac{At}{Ao}\times 100$$

where “*Ao*” indicates the initial wound dimension and “*At*” indicates the wound dimension at different days’ intervals.

#### 2.10.2. Histopathological Examination

Half of the rats were sacrificed by following the AVMA guidelines at the mid and remaining half at the end of the wound healing trial, and skin samples from each group that exhibited the entire wound, together with nearby normal skin, were carefully taken out for histology. The tissues were fixed in a 10% *v*/*v* formalin buffer solution, processed, and blocked with paraffin before being sectioned (4–6 µm) using a microtome. Histopathological investigation of the tissues was carried out using two different staining techniques, namely Masson’s trichrome staining and hematoxylin and eosin (H&E) staining, in order to evaluate the extent of wound healing in each group. Subsequently, slides were examined using a microscope (Accuscope 3000, Commack, NY, USA, CaptaVision 5.1) with a 5-megapixel camera [34].

#### 2.10.3. Estimation of Inflammatory Markers

At the middle and end of the study, rats’ blood was drawn from all testing groups to measure serum anti-inflammatory mediators like IL-6, TNF-α, P53, VEGF, and Annexin-A5. Each group’s blood was collected using disposable, non-endotoxin collecting tubes, and after waiting for the blood samples to clot, the samples were centrifuged at 6000 rpm for 30 min to collect serum. The expression of the cytokines (IL-6, TNFα, P53, VEGF, and Annexin5) was then assessed using the ELISA assay, for which 100 µL of serum sample was added to each well in ELISA microtiter plate and incubated for 90 min at 37 °C. After that, we removed the liquid, added 100 µL of Biotinylated Detection Ab/Ag, and further incubated it for 1 h at 37 °C. Then, it was aspirated and washed three times. After that, 100 µL of HRP conjugate was added, and it was incubated for 30 min at 37 °C, aspirated and washed five times, followed by addition of 90 µL of substrate reagent, and it was incubated again for 15 min at 37 °C. In last step, 50 µL of stop solution was added, and OD values were determined in ELISA microplate reader at 450 nm immediately. Results were calculated using absorbance values [3].

#### 2.10.4. Statistical Analysis

Each experiment was conducted at least three times, and the results were reported as an average. Two-way analysis of variance (ANOVA) and the *t*-test were used, followed by Tukey’s test for statistical evaluation. Results were considered statistically significant if the *p*-value was less than 0.05.

## 3. Result and Discussion

### 3.1. Selection and Optimization

During wound healing, maintaining a balance between long-term anti-inflammatory and short-term antibacterial treatment is extremely difficult. To tackle this issue, we developed a multifunctional bilayer film that releases the medication and essential oils sequentially, giving it antibacterial and antioxidant qualities. The current research is primarily concerned with the selection of an optimized formulation for treating wounds. Antibacterial assays of both the drug- and the essential oil-loaded films and the antioxidant activity of both films and oils were the main aspects considered when selecting the formulation. The final formulation included drug and oil concentrations with a higher inhibitory activity against Gram-negative (*E. coli*) and Gram-positive (*S. aureus*) bacteria. Other features, including thickness, weight variation, folding endurance, and swelling index were evaluated in this study to determine their potential in an optimized formulation.

### 3.2. Pre-Formulation Studies (Single-Layer Films)

#### 3.2.1. Physical Examination

Figure S2 (Supplementary Section) displays the visual representation of the prepared films. The films were transparent and clear when they were blank; when the formulation contained drugs and oil, they were semi-transparent and had a smooth texture. Additionally, they had no pores, fractures, or bubbles on their surface and were flexible, as previously observed [35].

#### 3.2.2. Thickness and Weight Variation

The thickness of the film typically depends upon the preparation technique, the amount of polymeric solution added to the dish, and the level of the surface throughout the drying process. For this reason, films of uniform thickness were produced by filling the petri dishes with predefined amounts of film-forming polymeric solution and drying them on a flat surface. Table 5 shows a gradual increase in film thickness from blank films to optimized bilayer films. The addition of the drug and essential oils was accountable for this gradual increase in film thickness because the solid content of the films rose [36]. The thickness of blank, optimized drug- and oil-loaded, and optimized bilayer films was in the range of 0.024 mm to 0.202 mm, as shown in Table 5. The weight of the films increased as the amount of oil and drug increased, similar to the thickness of the films. Because it contains both drug and oil content, the optimized bilayer film weighs more due to the presence of solid content, which raises the weight [37].

#### 3.2.3. Folding Endurance

A film’s ability to tolerate rupture is referred to as folding endurance. Table 6 shows the folding durability of drug-loaded, oil-loaded, and bilayer films. Oil-loaded and bilayer films had values that were more than 200 times higher. Perhaps as a result of the plasticizer component, the higher folding values offered a helpful indication of the film’s elasticity and flexibility [35]. Drug-loaded films have been shown to have lower endurance values since the polymer used to make them disintegrates quickly [38].

#### 3.2.4. Disintegration Time

The disintegration time for blank, drug-loaded, and optimized bilayer films was measured, as shown in Table 7. There is no discernible difference between the disintegration times of blank and drug-loaded films. Bilayer films developed by the double solvent casting technique had a longer disintegration period than single films. It takes roughly two to four times longer due to the adjacent layers in a bilayer film [39]. Furthermore, cross-linking on the primary layer influences and lengthens the secondary layer’s disintegration period in bilayer films [40].

#### 3.2.5. Swelling Index

**Figure 2:**
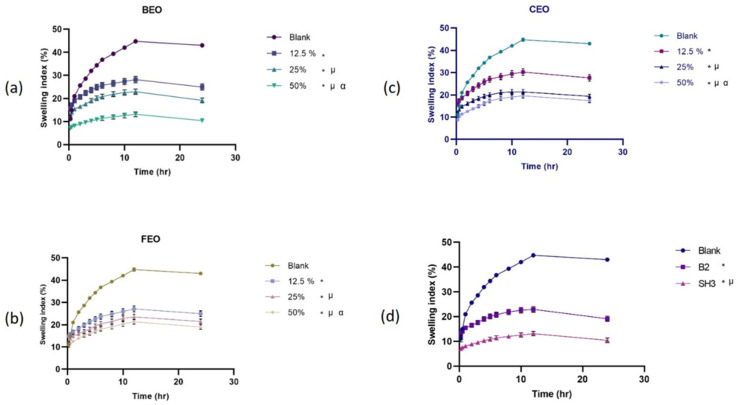
Swelling behavior of (**a**) bergamot oil-loaded films, (**b**) chamomile oil-loaded films, (**c**) frankincense oil-loaded films, (**d**) comparison of blank, oil film with optimized concentration, and optimized bilayer film (*n* = 3, *p* < 0.05, * when compared to blank, µ when compared to 12.5%, α when compared to 25%, and in (**d**) * when compared to blank, µ when compared to B2, α when compared to SH3).

The extent of swelling indicates the film’s water retention capacity. The swelling behavior of blank Na-Alg films, the optimized final bilayer film, and Na-Alg films loaded with different quantities of essential oils is shown in Figure 2. The findings showed that the higher degree of swelling in the blank film reduces as essential oils are added at various concentrations. The capacity to absorb water is attributed to the hydrophilic groups of the polymers in the film. Hydrogel films’ hydrophilic qualities are essential for preventing the accumulation of exudates discharged by wounds [41]. The prepared hydrogel films were found to have the potential to be used as a bandage for wounds. The films’ capacity to inflate is significantly influenced by the hydrophilicity of its constituents. The hydrogel films’ water-retention capacity declines as the hydrophobic oil content rises with the addition of essential oils; less hydrophilic components provide greater moisture resistance than more hydrophilic ones [42].

### 3.3. Antibacterial Assay

The colonization of wounds by various bacteria leads to infection and impedes the natural healing process [43]. Although topical antibiotic treatment can detain these harmful germs, overuse can cause bacteria to become resistant and perhaps endanger human health. Therefore, wound dressings require multiple antibacterial components to battle the wide spectrum of bacteria that can infect wounds and avoid the development of antibiotic resistance. Wound dressings that combine multiple antibacterial agents can provide a broad spectrum of antimicrobial activity, potentially improving wound healing outcomes [44].

An antibacterial assay of all the primary and secondary film formulations was conducted against Gram-positive (*S. aureus*) and Gram-negative (*E. coli*) bacteria in order to identify the optimal drug and oil concentration and select essential oils for the final bilayer formulation. The diameter of the zone of inhibition (ZOI) was measured and recorded in Table 8.

As shown in Figure S5 (Supplementary Section), 25% BEO has a higher zone of inhibition in the case of *E. coli* and a slightly higher zone in the case of *S. aureus*, demonstrating that bergamot essential oil produces the highest inhibition against bacterial growth out of the three essential oils. Similarly, Sánchez et al. have also investigated the antibacterial properties of bergamot oil, and the results against these two microorganisms are comparable [45]. The phytochemicals in BEO, such as limonene and linalyl acetate, are credited for its antibacterial activity [46]. The antibacterial activity of frankincense oil was not predominantly strong, although chamomile oil showed a mild antibacterial effect against both Gram-positive and Gram-negative microorganisms. The antibacterial qualities of essential oil-loaded films for wound healing were also assessed by Liakos et al., revealing that chamomile oil has no discernible growth suppression action against *S. aureus* and *E. coli* [47]. Frankincense oil (FEO) showed very low growth suppression action against *S. aureus* and *E. coli*, according to the findings of another study on the antimicrobial activity of FEO and other oils [48]. Additionally, data revealed that oils’ growth-inhibiting activity decreased with increasing concentrations. This could be attributed to the concentration of polymer, which may not be adequate for higher oil loadings [49].

Bacterial activity in drug-loaded films showed that the ZOI of 2.5% and 5% drug-loaded films differed somewhat, whereas the measured zone of inhibition did not significantly differ between 5% and 10% (Figure S6, Supplementary Section). Since the 5% drug-loaded films’ inhibitory capacity was comparable to 10%, 5% drug-loaded films were selected for further studies. In addition, 10% of the drug-loaded films showed some precipitation upon physical inspection. As reported in the literature by other authors, the gel matrix of polymers may restrain the diffusion of active substances such as drugs and oils at higher concentrations, which may be responsible for the abating inhibitory effect at a 10% drug concentration [50]. Similarly, it was reported by Ali and Blunden that physiochemical factors like solubility and diffusion may account for stagnant antibacterial activity even at higher concentrations [51].

### 3.4. Antioxidant Assay

**Figure 3:**
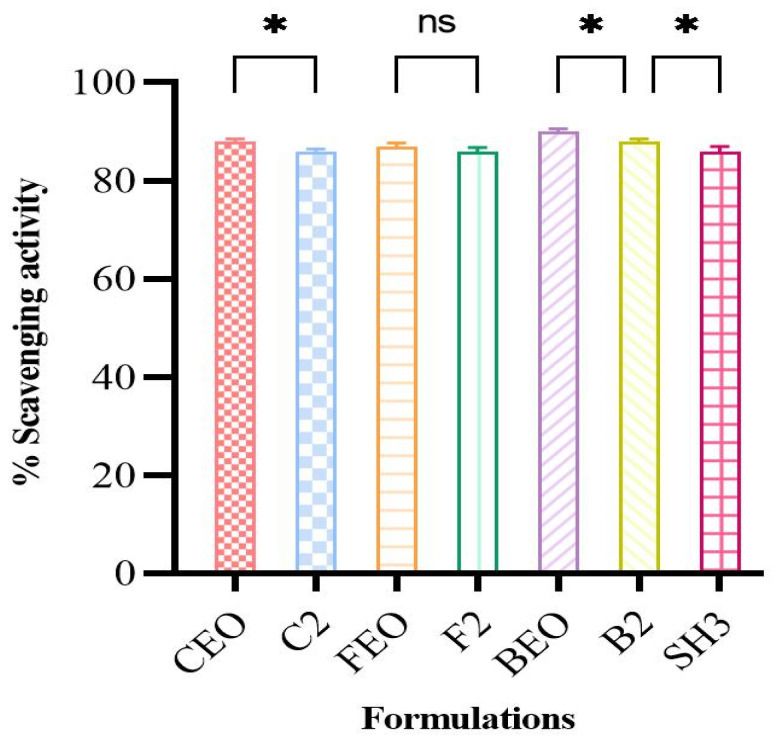
DPPH scavenging potential of pure oils, oil-loaded films, and bilayer film (*n* = 3 * if *p* < 0.05, ns if *p* > 0.05).

The ability of optimized oil films, bilayer films, and pure oils to scavenge the DPPH free radical and transform the stable DPPH into the reduced form was used to determine their free radical scavenging activity. According to the results (Figure 3), the percentage scavenging activity of pure oils varies only slightly after being incorporated into the films, suggesting that the oils retain their antioxidant activity after being incorporated into the polymeric matrix. The slight variation may be due to the crosslinking of the films [52]. Since a large number of free radicals at the wound bed prevent the damaged tissues’ natural healing process, the presence of antioxidant molecules in the hydrogel matrix aids in wound healing. The IC50 value is frequently used to compare the ability of various antioxidants to scavenge radicals. The IC50 values provide a quantitative description of the affinity for radical scavenging. These factors make the IC50 value one of the most valuable methods for assessing the DPPH radical scavenging tendencies. For BEO, the predicted IC50 was 4.2 ppm. The IC50 values for CEO and FEO were 173.1 and 16.40, respectively. BEO was shown to have a high antioxidant capacity because it has been documented that the lower the IC50 value, the stronger the antioxidant activity [53]. The antioxidant qualities of the BEO are demonstrated by the phenolic components, which efficiently block and remove free radicals from reactive oxygen species [54].

The antioxidant activity of oil-loaded films was also assessed with the aim of comparing them with pure oils, and it was seen that the overall scavenging activity of oils was conserved after being encapsulated in the film matrix. Statistical analysis also displayed the small difference (*p* > 0.05) in the scavenging potential of pure oils and film formulations, except for the FEO-loaded film, further increasing the antioxidant potential of our films.

After carefully evaluating the preformulation parameters of all the films, i.e., physical parameters, swelling index, antioxidant activity, and antibacterial activity, only D2 and B2 films were selected for bilayer formulations. Thus, only BEO-containing bilayers were chosen for further studies.

### 3.5. Solid State Characterization

#### 3.5.1. FTIR Analysis

**Figure 4:**
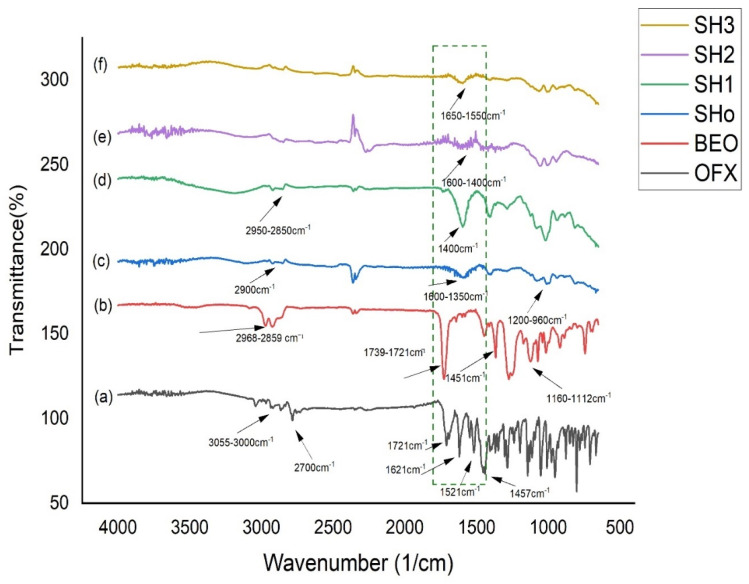
FTIR spectrum of (**a**) pure OFX, (**b**) BEO, (**c**) blank bilayer “SHo”, (**d**) oil-loaded bilayer “SH1”, (**e**) drug-loaded bilayer “SH2”, and (**f**) oil- and drug-loaded bilayer “SH3”.

Infrared spectrum (IR) analysis is a powerful technique for identifying the bonding and different functional groups present in a sample by looking at the vibrational energy levels of the molecules, which are basically the fingerprints of different molecules. FTIR analysis was used to confirm the interactions between the drug and the polymeric matrix (Na-Alg/HPMC+HEC bilayer film). The FTIR spectra of pure OFX, oil, and bilayer films are shown in Figure 4. Since each band in the bilayer film indicates the presence of a functional group in the ingredients, the FTIR chemical interaction studies showed that there was no chemical reaction between the drug, oil, and excipients.

The chemical stability of OFX was verified by an analysis of the spectrum data. OFX displayed bands at 1621, 1548, 1521, and 1457 cm^−1^, which were caused by aromatic C = C stretching vibrations, and a broad band at 1712 cm^−1^, which was attributable to the C = O stretching vibrations [55]. Moreover, the methyl (CH3) groups are responsible for the peaks seen at 2700 cm^−1^, while the stretching peaks observed at 3055 cm^−1^–3000 cm^−1^ are caused by the vibration of the hydroxyl group and the existence of intermolecular hydrogen bonding in the OFX structure [28]. The huge peaks in the pure BEO spectra, which were seen in the 2968–2859 cm^−1^ area, were ascribed to the asymmetric stretching of C–H. There were noticeable sharp peaks at 1739 and 1721 cm^−1^, which indicated that the (C = O) carbonyl group stretching. Alkene or aromatic carbon double bonds (C = C) with wave numbers ranging from 1671 to 1643 cm^−1^ were detected in the BEO spectra. Wave numbers 1451–1411 cm^−1^, 1160–1112 cm^−1^, and 1019 cm^−1^ were shown to originate from the C–H bending, C–O stretch, C–C stretch, and C–N aliphatic stretches, respectively. The stretching of the aromatic C–H (benzene ring) was also responsible for the absorptions in the 919–689 cm^−1^ range, and the out-of-plane bending was caused by the asymmetric stretching of the C–H [56].

The third spectrum of the blank bilayer film (SHo) represents the peaks of polymers used for film formation, such as Na-Alg and HPMC. The stretching vibration of the carboxylic group was found in the 1600–1350 cm^−1^ range. Furthermore, it was previously established that the absorbance bands at 1200–960 cm^−1^ were responsive to the skeletal vibrations of the six-membered (pyranose) alginate ring [57]. At 2900 cm^−1^, the symmetric stretching mode of hydroxypropyl groups was discovered, wherein every C–H bond undergoes phase changes of extension and contraction. This peak is attributed to HPMC [58]. The spectra of the bilayer film that was solely loaded with oil (SH1) showed that, with a minor shift brought on by the polymeric effect, it kept the oil peaks that were previously assigned. Accordingly, only the drug-loaded bilayer film (SH2) displayed overlapping polymeric peaks that were located in the same location as the drug [59]. Film loaded with the oil and drug concurrently showed the shifting of peaks in (SH3) due to a polymeric interaction with the drug and oil, and other broader peaks of the spectra are maintained, showing the merging of drug and oil peaks in polymeric peaks [60].

#### 3.5.2. Scanning Electron Microscopy

**Figure 5:**
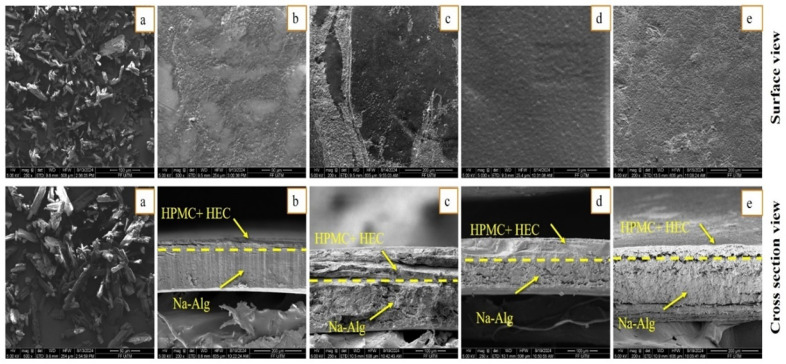
SEM micrographs of (**a**) pure OFX, whereas (**b**) blank bilayer film “SHo”, (**c**) oil-loaded bilayer film “SH1”, (**d**) drug-loaded bilayer film “SH2”, and (**e**) oil and drug loaded film “SH3” exhibits surface and cross section view.

Scanning electron micrographs of pure OFX, surface, and cross-section views of bilayer formulations (SHo, SH1, SH2, and SH3) are shown in Figure 5. Scanning micrographs of pure OFX exhibited a sharp, needle-like structure, which supports its crystalline nature, whereas a similar morphology has also been reported previously [61]. In the case of bilayer formulations (SHo, SH1, SH2, and SH3), the surface view showed a continuous, non-porous, slightly rough, and microstructured surface without any cracks, which indicates the good compatibility between the polymeric matrix of bilayer films; quite similar surface morphology has also been viewed by other authors [62].

The cross-sectional view of the blank bilayer film (SHo) revealed two distinct layers that were uniform, continuous, and had a compact lamellar appearance, suggesting that the polymeric matrix of both layers was well-miscible. In comparison, the non-cross-linked immediate layer, containing HPMC and HEC, was more compact, while the cross-linked sodium alginate layer was more porous or lamellar, as observed previously [17]. In the case of loaded formulations, Figure 5 presents the continuous and micro-morphology of the bilayer films’ outer layers, tightly bonded with the inner layer. This confirms the polymer matrix’s optimal interfacial adhesion and compatibility, as well as the outer layer’s function as an effective barrier to prevent the outward release of essential oil. Drug and oil addition produced a very distinct cross-sectional morphology with distinct inner and outer layers. As shown in Figure 5, the oil-containing inner layers displayed a more pronounced microporous structure, and no indication of the oil droplets’ separation from the polymer matrix was seen. This occurrence demonstrated that the oil was evenly distributed and well-emulsified within the film matrix and presents higher retention of oil. The thickness difference between the bilayer film mainly depends on the presence of the drug and oil in the polymeric matrix. The outer drug-loaded layer with a more consistent and diminutive microporous structure may indicate a stronger contact between the drug molecule and the polymer, resulting in a smaller spatial distance, leading to a smaller thickness [63].

#### 3.5.3. X-Ray Diffractometry

**Figure 6:**
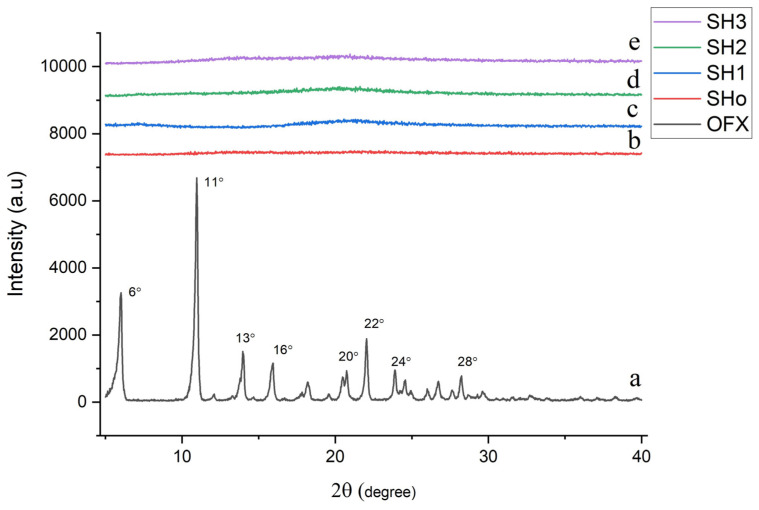
XRD graph of (**a**) pure OFX, (**b**) blank bilayer film (SHo), (**c**) oil-loaded bilayer film (SH1), (**d**) drug-loaded bilayer film (SH2), and (**e**) oil- and drug-loaded bilayer film (SH3).

The crystalline or amorphous nature of a sample is determined by X-ray diffraction. The X-ray diffractogram of OFX, blank bilayer film, and bilayer films filled with drug and oil are displayed in Figure 6. OFX’s crystalline nature was corroborated by the results, which showed prominent peaks at 2θ = 6°, 11°, 13°, 16°, 20°, 22°, 24°, and 28°, as supported by the literature [61]. However, the diffractogram of bilayer films loaded with both the drug and oil showed no noticeable peaks, suggesting that the formulation is amorphous and that the drug and oil have been fully dispersed into the polymeric matrix [64].

#### 3.5.4. Thermogravimetric Analysis

**Figure 7:**
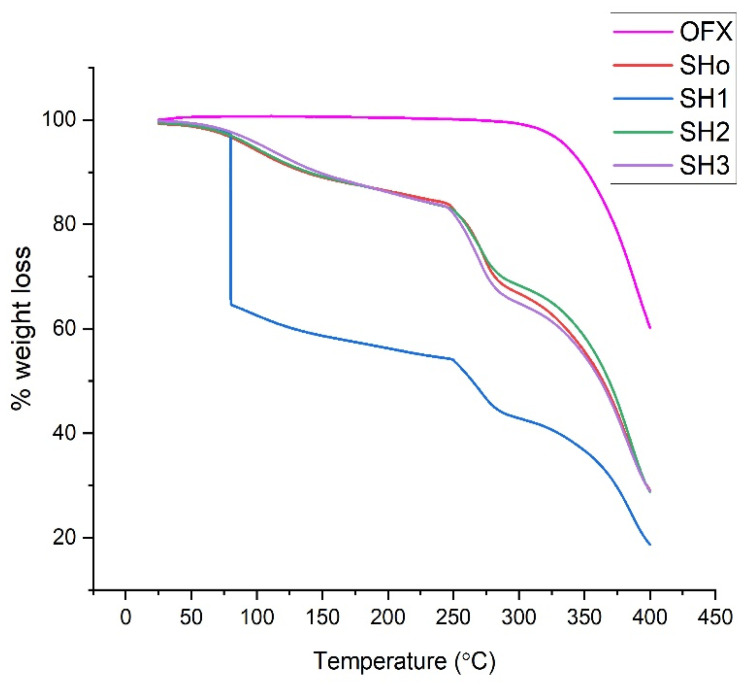
TGA thermograms of pure OFX, blank bilayer film (SHo), oil-loaded bilayer film (SH1), drug-loaded bilayer film (SH2), and oil- and drug-loaded bilayer film (SH3).

Thermogravimetric analysis of pure drug (OFX), blank bilayer film (SHo), oil-loaded bilayer film (SH1), drug-loaded bilayer film (SH2), and oil- and drug-loaded bilayer film (SH3) was carried out to determine the formulation’s weight loss during heating, which establishes the stability of the samples (Figure 7).

The thermogram of pure OFX shows stability up to 270 °C. Afterwards, degradation starts. This phase ranges from 277–400 °C, and it shows sudden and maximum degradation at a temperature of 277 °C, corresponding to the melting point of OFX, as observed previously [28]. The weight loss during heating of the samples was analyzed to ascertain the thermal stability of the bilayer films. It was found that each sample’s breakdown trend was similar, with the exception of SH1, which exhibits a sudden huge weight loss between 50 and 100 °C and a steady weight loss between 250 and 400 °C. Due to the breakdown of the components, the weight decreased significantly as the temperature increased in the 250–350 °C range. The process of weight loss and evaporation reduces the moisture content of the film, resulting in the first step of weight loss. The second stage of weight loss could be brought on by the breakdown of polymer structures [65].

#### 3.5.5. Differential Scanning Calorimetry

**Figure 8:**
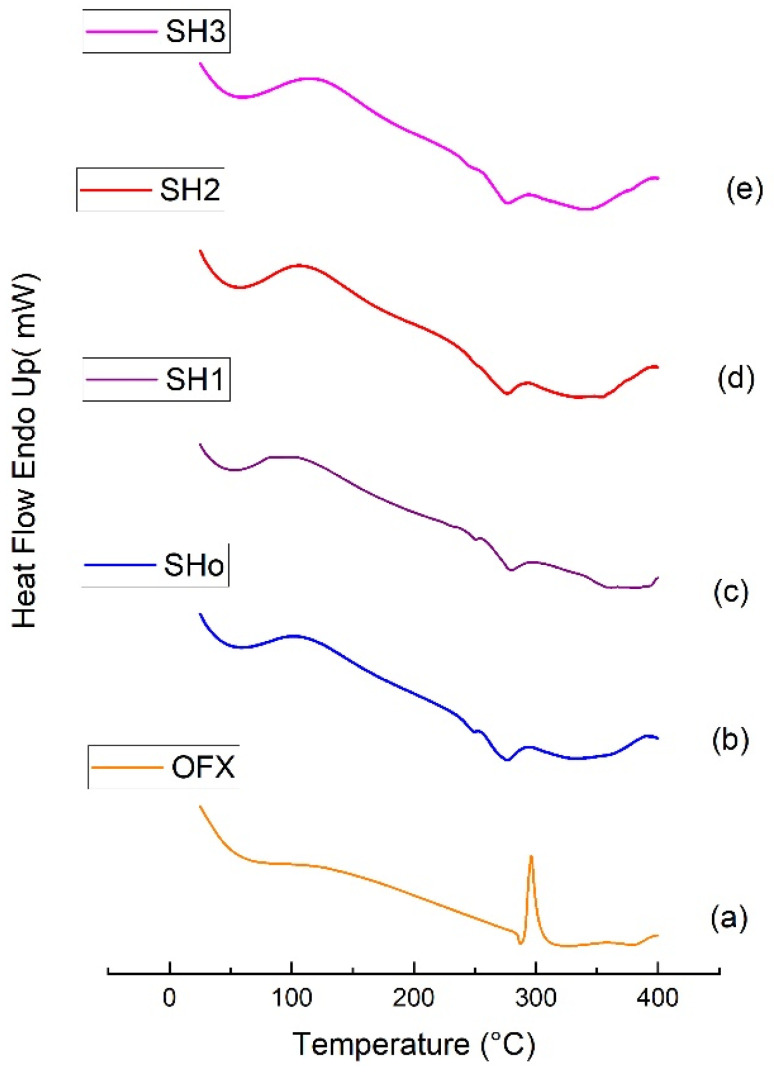
DSC thermograms of (**a**) pure OFX, (**b**) blank bilayer film SHo, (**c**) oil-loaded bilayer film SH1, (**d**) drug-loaded bilayer film SH2, and (**e**) oil- and drug-loaded bilayer film SH3.

Pure drug (OFX), blank bilayer film (SHo), oil-loaded bilayer film (SH1), drug-loaded bilayer film (SH2), and oil-and drug-loaded bilayer film (SH3) thermograms obtained by differential scanning calorimetry are shown in Figure 8. Water evaporation and the accompanying loss of bound water were the causes of the broad endothermic event that was noticed in the thermogram of pure OFX that occurred between 50 and 90 °C [66]. At 277 °C, another exothermic peak appeared, indicating the OFX’s melting point. A high degree of stability is indicated by a high crystalline melting temperature [40]. Bilayer film thermograms revealed broad endothermic peaks at two distinct locations, signifying two distinct melting phases. The first endothermic peak appeared between 30 °C and 76 °C, and the second melting phase took place between 240 °C and 274 °C [67]. Water loss is shown by the first melting phase in the films’ mass loss profile, whereas peaks at 200–250 °C show the breakdown of polymers and plasticizer. Nevertheless, the films continue to decompose, albeit more slowly, after the temperature at which the polymers begin to degrade [68].

### 3.6. Drug Content Determination

To determine how much drug was entrapped, the percentage drug loading and entrapment of optimized bilayer formulation SH3 were calculated. The calculated entrapment and loading efficiency for drug and oil were 99.3% and 98.85%, respectively, which shows that the film has high drug content with minor losses (*n* = 3). This shows that the drug and oil were dispersed uniformly throughout the films and that the preparation techniques could yield films with a uniform composition [36].

### 3.7. In Vitro Drug Release

**Figure 9:**
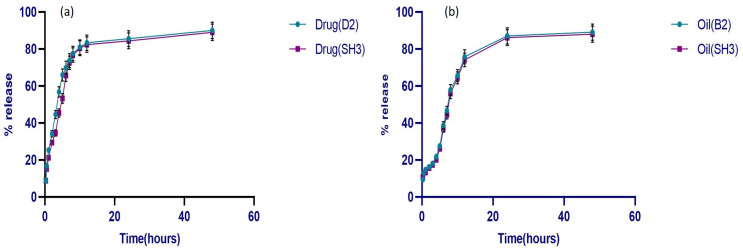
Release behavior of (**a**) drug from D2 and SH3, (**b**) oil from B2 and SH3 (*n* = 3).

The final optimized formulation SH3 (drug- and oil-loaded bilayer film) was compared with the drug release behavior of formulations D2 (just drug-loaded film) and B2 (only oil-loaded film). The results of the release studies (Figure 9) showed that over 80% of the drug and oil were released from both single and bilayer films, whereas there was a slight decrease in the drug and oil release from the bilayer film over a period of more than 48 h.

Initially, the drug from both single and bilayer films showed an abrupt release of over 50% in the first four hrs, followed by a progressive reduction after 12 h. The drug’s release profile was biphasic, meaning it happened in two stages at once. There was a brief initial phase called burst release, followed by the second, slower release phase. A polymeric film matrix’s burst release phase is determined by the rate at which the solvent dissolves the drug molecules adsorbed on its surface [69]. Since there is no erosion effect on the films during the drug release in a Franz cell, the drug release from the primary layer following the burst release phase exhibited sustained behavior over time. Therefore, the full release took longer than the primary layer’s disintegration duration [70]. On the other hand, because the oil-loaded film contained both hydrophilic and hydrophobic components, the oil release behavior was not abrupt and was only 20% within the first four hrs. It is anticipated that hydrophobic substances, such as lipids present in oil, such as limonene, linalool, and terpenes, may enhance the controlled release behavior and act as a barrier to stop abrupt or quick release from the film. On the other hand, the presence of the hydrophilic polymer permits water molecules to flow through the film. Apart from its therapeutic properties, oil gives films their hydrophobicity, which means it can impede release from the polymeric matrix [71]. Statistically, there is no significant difference in drug release from single and bilayer films except at certain time points, where the drug from the SH3 formulation showed a relatively slower release. While oil release had no significant differences in single and bilayer films, the crosslinking of the primary film may be the cause of a modest decrease in drug release from the bilayer film. As crosslinking has a direct correlation with release behavior, oil release from the B2 formulation and bilayer film was also the slowest at first. Hence, the low swelling index of the oil-loaded and the bilayer films results in reduced release from these crosslinked formulations [72].

### 3.8. Release Kinetics

Four distinct kinetic models were fitted to the in vitro release data of the drug from bilayer films, as shown in Table 9. The R^2^ value of the Korsmeyer–Peppas model was 0.9899 and was the best-fit model for films with the highest drug loading [73]. The drug release mechanism is represented by the n value extracted from the Korsmeyer–Peppas model. The n value was determined to be > 0.5, and the non-Fickian drug release behavior was indicated [74]. When 0.5 > n < 1, it implies a non-Fickian (Anomalous) release case Ι, i.e., both diffusion and relaxation of the polymer chain. Different authors observed comparable release processes for various drugs and essential oils for hydrogel films [75].

### 3.9. In Vivo Assays

#### 3.9.1. Wound Healing

To evaluate the synergetic wound healing ability of bilayer films, wound contraction percentages were compared to controls using SHo (blank bilayer), SH1 (oil loaded bilayer film), SH2 (drug loaded bilayer film), and SH3 (oil and drug loaded bilayer film) over time (0, 3, 6, 9, and 12 days) in a rodent wound model.

On day 3, considerable contraction was seen in all formulations compared to the control and results (Table 10). The highest contraction was observed in group F, which received the formulation SH3, containing both oil and drug in adjacent layers. Formulation SH1, containing oil, also showed significant wound contraction, slightly less than SH3. SH2 and SHo formulations exhibited wound contraction capabilities that were lower than previously mentioned formulations. This wound contraction trend was also noticed on the 6th, 9th, and 12th days. At the end of the study, maximum wound contraction (98.5%) was observed in the group receiving the SH3 formulation due to the synergistic activity of both the oil and drug from the adjacent layer of bilayer films, as compared to the control and other groups receiving the oil and drug separately. It shows that the antibacterial activity of the drug and the anti-inflammatory and antioxidant activity of the oil help to promote healthy wound healing synergistically [33].

**Figure 10:**
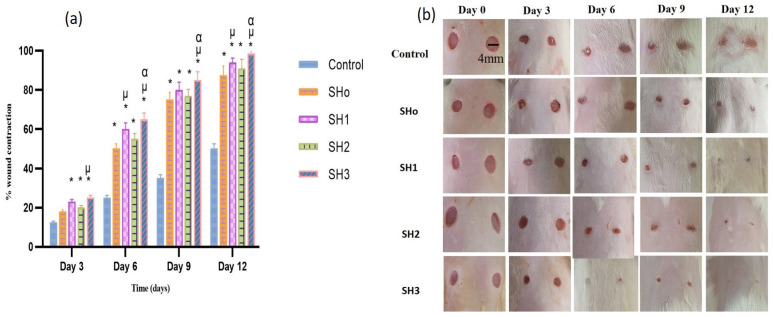
(**a**) Comparison of wound healing potential for different formulations and stages of wound reduction at several days (*n* = 6, *p* < 0.05) * when compared to control, µ when compared to SHo, α when compared to SH2, (**b**) visual images of wound treated with different treatment groups on days 0, 3, 6, 9 and 12.

In the case of comparison between disease control and treatment groups, a significant difference (*p* < 0.05) in the rate of wound contraction was noticed (Figure 10). The improvement in wound healing after the application of a blank bilayer (SHo) film was attributed to the structural features of the hydrogel and supporting humid conditions that back wound healing [76]. The group treated with the SH3 formulation manifested statistically significant (*p* < 0.05) wound contraction ability, while in the case of treatment groups D and E, which received the SH1 and SH2 formulations, respectively, there was no statistically significant difference (*p* > 0.05) in wound closure rate. This proposes that both BEO and OFX showed comparable results in terms of wound healing. Moreover, it was also suggested that the wound healing properties of oil were comparable to those of the antibacterial drug. These results were in accordance with [77]. Research suggests that the primary components of BEO, such as limonene, linalool, and linalyl acetate, have demonstrated that their anti-inflammatory, immunomodulatory, and preventive skin damage properties are behind its significant wound healing properties [78].

#### 3.9.2. Inflammatory Marker Estimation

Pro-inflammatory cytokines, including IL-6, TNF-α, and other chemokines, are essential for drawing cells for debris removal and growth factor recruitment during the early phases of wound healing [79]. For the best possible wound healing, this early inflammation must be carefully controlled and promptly resolved. We estimated five different biomarkers, IL-6, TNF-α, VEGF, P53, and Annexin-A5, during and at the termination of wound healing activity.

TNF-α and IL-6 are part of the “inflammatory cascade”, which is the successive stimulation of immune response pathways [80,81]. As part of their inflammatory response, macrophages release TNF-α and IL-6 when they identify inflammation or infection, stroke, and cardiovascular illness [82,83]. Bilayer films containing both the oil and drug were able to reduce the levels of markers, indicating a resolution of inflammation and improvement in wound healing [84]. Angiogenesis is a vital phase in wound healing, and failure causes persistent wounds. The concept of restoring blood flow to damaged tissues by stimulating neo-angiogenesis has been extensively investigated. Vascular endothelial growth factor (VEGF), an important dynamic molecule in angiogenesis, is an emerging therapeutic target for wound healing strategies [85]. As a skin wound heals, differentiated myofibroblasts and recruited fibroblasts multiply and deposit extracellular matrix (ECM) to produce the granulation tissue. Later on, in the healing process, myofibroblasts are forced into senescence. Senescence in cutaneous wounds is regulated by the matricellular protein CCN1, also called CYR61, which is dynamically expressed at the sites of wound healing and inflammation. p53 is activated by CCN1, which causes a far greater and longer-lasting level of ROS, which causes tissue damage and hinders cell growth, hence, compromising the healing process. This upregulation of ROS (reactive oxygen species) is regulated by p53, and limiting its level has a beneficial effect on wound healing [86]. Annexin A5 promotes wound healing and inflammation regulation by causing macrophages to shift from the M1 to M2 phenotype. Annexin A5 significantly reduces pro-inflammatory factors, strongly promotes epithelial cell migration, and contributes to cell membrane healing [87].

**Figure 11:**
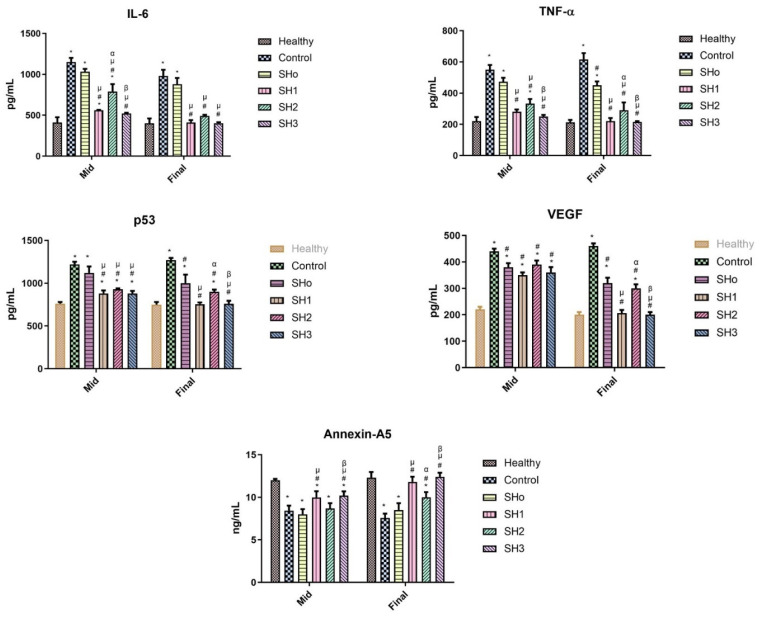
Levels of inflammatory markers (IL-6, TNF-α, p53, VEGF, and Annexin-A5) observed in the rats’ serum of different groups at the mid and end of wound healing study. (*n* = 6, *p* ˂ 0.05 * when compared to healthy, # when compared to control, µ when compared to SHo, α when compared to SH1, β when compared to SH2).

Figure 11 reveals that mid-study, the group treated with SHo (blank formulation) had a non-significant difference when compared to the control group, and the other groups treated with formulations SH1, SH2, and SH3 had a significant difference in levels of IL-6 and p53 compared to the control group, but were still higher than normal levels, indicating that inflammation did not diminish completely. While TNF-α typically subsides earlier than IL-6, as statistically proven in the middle of the activity [88]. At the end of the wound healing activity, when the wound was healed, the level of these inflammatory markers declined as the inflammation subsided, and the levels of these inflammatory markers were similar to those of the healthy group. The upregulation of p53 mid-study was prominent due to higher levels of ROS. Later, at the final stages of wound healing, the level of p53 went down as inflammation subsided due to inhibition of the ROS, and the epithelial lining of skin tissues was restored as the skin tissues regenerated [89]. In the case of VEGF, mid-study, there was no significant difference in the levels of VEGF in the control group and treatment groups except for SH1, as the angiogenesis process was just starting (after three or four days of injury, capillaries become visible in the wound bed). The final stages of healing involved the suppression of angiogenesis. When inflammation diminished, the levels of VEGF in the treated groups became equal to those of the healthy group. Growth factors decrease in the wound as tissue hypoxia is restored and inflammation goes down [90]. In the case of Annexin-A5, the results showed that mid-study, the blank and only drug-treated groups had Annexin-A5 nearly equal to the control group, indicating an impaired cell membrane, while SH1 and SH3 treatment groups showed healing to a certain extent. At the terminal stages of wound healing, SH1- and SH3-treated groups have gained the same normal level of Annexin-A5 as that in the healthy group, and the SH2-treated group showed cell membrane repair to a lesser extent compared to other treatment groups. Annexin-A5 is well-known for its pro-apoptotic and anti-inflammatory characteristics. It has been suggested that Annexin accumulation at the wound site promotes wound healing by limiting wound expansion. Elevated Annexin-A5 levels in treatment groups relative to control may be indicative of the reduced inflammation, cell membrane repair, and healing phase of wound maturation [91].

#### 3.9.3. Histological Examination

The effects of each bilayer dressing on wound healing were examined in the histological assessment of in vivo investigations, which sought to compare the wound healing performance of bilayer films with control and healthy groups. For this purpose, on days 6 and 12, cross-sections of tissue samples taken from the wound areas covered by each dressing were examined.

**Figure 12:**
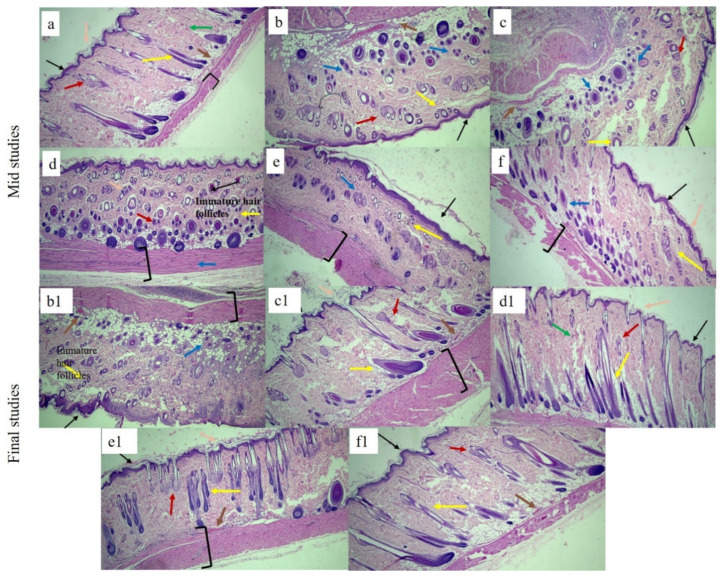
H&E-stained histological micrographs of (**a**) healthy, (**b**,**b1**) control, (**c**,**c1**) SHo, (**d**,**d1**), SH1, (**e**,**e1**), SH2, and (**f**,**f1**) SH3 bilayer film at 10X at mid and terminal stages, respectively. Black arrow indicates epidermis, blue indicates macrophages, pink indicates stratum corneum, brown indicates blood capillaries, green indicates collagen fibers, red indicates sebaceous glands, and yellow indicates hair follicle brackets for connective tissues.

Figure 12 displays the tissue micrographs on the sixth day post-incision. It was discovered that although the wound sites had not fully epithelialized, inward epithelial development continued. Additionally, it was observed that epithelization began randomly beneath the wound crust (patch type). There was modest to moderate enhanced vascularization and fibrosis in every picture. Immature hair follicles were also seen. Furthermore, the development of granulation tissue was noted, which is defined by the build-up of macrophages and some non-specific inflammatory cells. A similar type of finding for mid studies was also reported elsewhere [92].

At the terminal stage, healed wound tissues were carefully examined in order to verify the coherence of the regenerated epidermis, dermis, and related structures, such as hair follicles, blood vessels, inflammatory cells, and sebaceous glands, among different groups. Histograms. Figure 12 showed that the control group still had a large number of macrophages and inflammatory cells; no mature hair follicle was seen due to impaired healing. Also, complete epithelialization could not be seen in the control group. The wounds treated with the bilayer formulations (SHo, SH1, SH2, and SH3) indicated newly formed hair follicles, and the epidermis was also visible, along with negligible inflammatory cells in fewer groups. In the group treated with SH3, the epidermis and dermis remained nearly intact, with decreased epithelial thickness, a sign of wound healing. The SH3 group demonstrated an extensive network of newly formed blood vessels, improved epithelialization, and a greater rate and extent of healing, all of which suggest full wound healing. The increased anti-inflammatory action of the bilayer film was attributed to the absence of inflammatory cells in the SH1 and SH3 groups [93].

**Figure 13:**
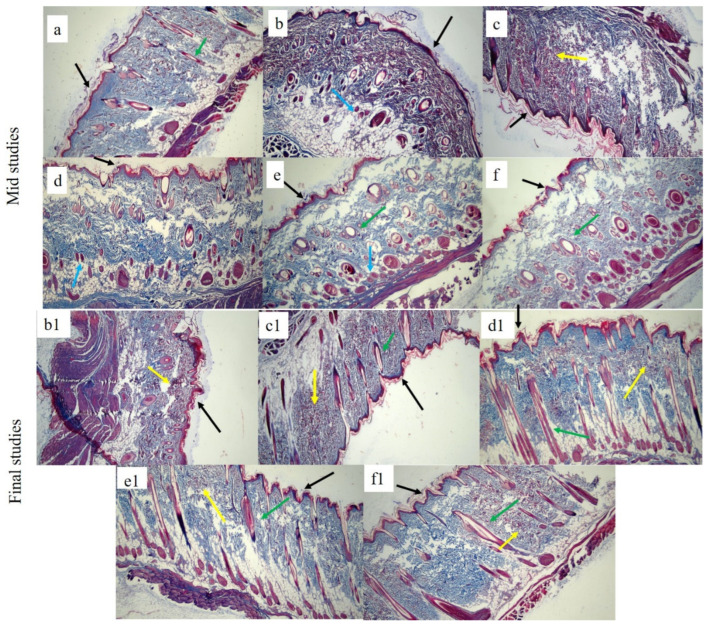
Masson trichome-stained histological micrographs of (**a**) healthy, (**b**,**b1**) control, (**c**,**c1**) SHo, (**d**,**d1**) SH1, (**e**,**e1**) SH2, and (**f**,**f1**) SH3-treated groups at 10X. Black arrow indicates the epidermis, blue indicates macrophages, green indicates hair follicles, and yellow indicates collagen fibers.

To demonstrate collagen deposition and its organization in the skin, Masson’s trichome was used, as seen in Figure 13. Even on day twelve, the collagen fibers in the control and SHo groups were loosely packed. However, in the SH2 groups, dermal collagen deposition was better than in the control group. Nonetheless, the SH1 and SH3 groups showed tightly packed bright blue collagen fibers that were almost identical to those found in normal skin. The images also clearly show that the newly created dermis had more organized collagen deposition and a larger collagen-occupied area than the other groups [94]. In this study, the clinical decrease in redness is correlated with the normalized collagen arrangement and decreased inflammatory cell presence seen in Figure 12 and Figure 13. This implies that the therapy fosters a more controlled wound healing response by modifying the underlying pathological processes, such as excessive angiogenesis and persistent inflammation, in addition to improving the macroscopic look of scars (hypertrophic scars). As a result, the histological results support the notable improvement in redness as key evidence of scar remodeling and the effectiveness of treatment [95].

## 4. Conclusions

To conclude this research, we developed a transparent bilayer film with both short-term antibacterial and long-term anti-inflammatory properties to treat skin wounds and promote tissue healing. The optimized hydrogel-based bilayer films with OFX (37.5 mg) and a 25% *w*/*w* concentration of bergamot oil showed good mechanical strength, disintegration, and swelling. The optimized film formulations retained the antioxidant property of oil and the strong synergistic antibacterial activity of the drug and oil against *S. aureus* and *E. coli.* The in vivo results showed that OFX and bergamot oil work synergistically to fight inflammation and bacterial growth while preserving an environment that speeds up wound closure. Histopathological investigations indicated that rats treated with bilayer films rapidly healed due to epidermal regeneration, increased collagen density, and the downregulation of inflammatory markers. Our findings suggest that prepared bilayer films can offer antibacterial coverage, reduce inflammation, and stimulate reepithelialization, leading to better wound healing.

## Figures and Tables

**Table 1:** Formulations of primary layer (sustained release layer).

Code	Sodium Alginate (*w*/*v*%)	Oil (*w*/*w*%)	Plasticizer (*w*/*w*%)
SA_Blank_	2	-	20
B1	2	12.5	20
B2	2	25	20
B3	2	50	20
C1	2	12.5	20
C2	2	50	20
C3	2	50	20
F1	2	12.5	20
F2	2	25	20
F3	2	50	20

Here, formulation codes are as follows: B = bergamot oil, C = chamomile oil, and F = frankincense oil.

**Table 2:** Formulations of secondary film (immediate release layer).

Code	Polymer HPMC E5+ HEC (*w*/*v*%)	Drug [16]	Plasticizer (*w*/*w*%)
D_Blank_	2.5 + 0.5	-	15
D1	2.5 + 0.5	18.25	15
D2	2.5 + 0.5	37.5	15
D3	2.5 + 0.5	75	15

**Table 3:** Formulations of bilayer membrane.

Code	Drug [16]	Oil (BEO *w*/*w*%)
SHo	-	-
SH1	-	25
SH2	37.5	-
SH3	37.5	25

**Table 4:** Classification of rat groups for wound healing.

Groups					
Group A	Group B	Group C	Group D	Group E	Group F
Healthy	Control	SHo	SH1	SH2	SH3

**Table 5:** Thickness and weight variation measurement of films (*n* = 10).

Code	Thickness (mm ± SD)	Weight Variation ± SD (g)	Code	Thickness (mm ± SD)	Weight Variation ± SD (g)
SA_blank_	0.024 ± 0.002	0.016 ± 0.002	F3	0.088 ±0.001	0.031 ± 0.002
B1	0.058 ± 0.003	0.024 ± 0.003	D_blank_	0.084 ± 0.001	0.024 ± 0.001
B2	0.066 ± 0.002	0.028 ± 0.001	D1	0.102 ± 0.002	0.029 ± 0.004
B3	0.078 ± 0.004	0.032 ± 0.004	D2	0.114 ± 0.002	0.033 ± 0.001
C1	0.068 ± 0.003	0.025 ± 0.002	D3	0.124 ± 0.004	0.036 ± 0.003
C2	0.076 ± 0.005	0.030 ± 0.004	SHo	0.148 ± 0.002	0.048 ± 0004
C3	0.086 ± 0.004	0.032 ± 0.003	SH1	0.192 ± 0.003	0.056 ± 0.002
F1	0.064 ± 0.002	0.026 ± 0.003	SH2	0.188 ± 0.004	0.064 ± 0.003
F2	0.076 ± 0.003	0.029 ± 0.002	SH3	0.202 ± 0.003	0.070 ± 0.004

**Table 6:** Folding endurance of optimized single- and bilayer films (*n* = 10).

Code	Folding Endurance ± SD	Code	Folding Endurance ± SD
SA_blank_	250 ± 5	F3	275 ± 7
B1	270 ± 6	D_blank_	155 ± 8
B2	280 ± 8	D1	155 ± 6
B3	275 ± 7	D2	150 ± 7
C1	260 ± 6	D3	150 ± 8
C2	275 ± 8	SHo	270 ± 5
C3	270 ± 7	SH1	265 ± 7
F1	265 ± 6	SH2	260 ± 8
F2	270 ± 8	SH3	250 ± 6

**Table 7:** Disintegration time of drug-loaded and bilayer films (*n* = 10).

Code	Disintegration Time (min) ± SD
D_blank_	8 ± 1
D1	8 ± 1
D2	8 ± 1
D3	10 ± 1
SHo	12 ± 1
SH1	14 ± 1
SH2	14 ± 1
SH3	14 ± 1

**Table 8:** Comparison of antibacterial activity of films loaded with different concentration of drug and oil for optimization (*n* = 3).

Code	Drug/Oil (%)	Diameter of ZOI (mm) ± SD		Code	Drug/Oil (%)	Diameter of ZOI (mm) ± SD	
		*S. aureus*	*E. coli*			*S. aureus*	*E. coli*
D1	2.5% OFX	38 ± 1	42 ± 1	C1	12.5% CEO	6 ± 1	6 ± 1
D2	5% OFX	40 ± 1	44 ± 2	C2	25% CEO	11 ± 2	10 ± 1
D3	10% OFX	44 ± 2	50 ± 1	C3	50% CEO	8 ± 1	8 ± 1
B1	12.5% BEO	10 ± 0.5	10 ± 0.5	F1	12.5% FEO	12 ± 0.5	10 ± 1
B2	25% BEO	18 ± 1	16 ± 2	F2	25% FEO	12 ± 1	12 ± 1
B3	50% BEO	12 ± 0.5	13 ± 0.5	F3	50% FEO	10 ± 0.5	10 ± 0.5

**Table 9:** Correlation coefficient values for zero-order, first-order, Higuchi, and Korsmeyer–Peppas models.

Code	Zero-order	First-order	Higuchi Model	Korsmeyer–Peppas Model	
	R^2^	R^2^	R^2^	R^2^	*n*
D2	0.8714	0.9515	0.9665	0.9899	0.624
B2	0.9180	0.8860	0.8145	0.9197	0.932

**Table 10:** Percent wound contraction for formulation on different days.

Groups	Formulations	Percentage Wound Contraction			
		Day 3	Day 6	Day 9	Day 12
Group A	Control	12.5 ± 2.5	25 ± 2	35 ± 3	50 ± 2
Group B	Blank bilayer	18 ± 2	50 ± 3	75 ± 2	87.5 ± 2.5
Group C	Oil loaded bilayer	23 ± 2	60 ± 2	80 ± 3	95 ± 2
Group D	Drug loaded bilayer	20 ± 3	55 ± 3	78.5 ± 2.5	92 ± 2
Group E	Oil and drug loaded bilayer	25 ± 2	65 ± 2	85 ± 2	98.5 ± 1.5

## Data Availability

Most of the data is presented in the article. However, the raw or processed data that was required to reproduce these findings cannot be shared at this time due to technical or time limitations.

## Supplementary Materials

The following supporting information can be downloaded at: https://www.mdpi.com/article/10.3390/pharmaceutics17121589/s1, Figure S1: Optical image of disintegration of D2; Figure S2: Physical appearance of prepared films (a) blank film (SA blank) (b) oil loaded film (B2) (c) blank HPMC and HEC film (D blank) (d) drug loaded film (D2); Figure S3: Optical images of single layer films; Figure S4: Optical images of 1.5 × 1.5 cm^2^ patches of bilayer formulation; Figure S5: Comparison of antibacterial activity of film loaded with oils at different concentration against *S. aureus* and *E. coli*; Figure S6: Comparison of anti-bacterial activity films loaded with drug at different concentration against *S. aureus* and *E. coli.*

## References

[1] Zhong Y., Xiao H., Seidi F., Jin Y. (2020). Natural polymer-based antimicrobial hydrogels without synthetic antibiotics as wound dressings. Biomacromolecules.

[2] Atiyeh B.S., Hayek S.N., Gunn S.W. (2005). New technologies for burn wound closure and healing—Review of the literature. Burns.

[3] Contardi M., Summa M., Picone P., Brancato O.R., Di Carlo M., Bertorelli R., Athanassiou A. (2022). Evaluation of a multifunctional polyvinylpyrrolidone/hyaluronic acid-based bilayer film patch with anti-inflammatory properties as an enhancer of the wound healing process. Pharmaceutics.

[4] Mahmood H., Khan I.U., Asif M., Khan R.U., Asghar S., Khalid I., Khalid S.H., Irfan M., Rehman F., Shahzad Y. (2021). In vitro and in vivo evaluation of gellan gum hydrogel films: Assessing the co impact of therapeutic oils and ofloxacin on wound healing. Int. J. Biol. Macromol..

[5] Vowden K., Vowden P. (2017). Wound dressings: Principles and practice. Surgery.

[6] Thu H.-E., Zulfakar M.H., Ng S.-F. (2012). Alginate based bilayer hydrocolloid films as potential slow-release modern wound dressing. Int. J. Pharm..

[7] Luneva O., Olekhnovich R., Uspenskaya M. (2022). Bilayer hydrogels for wound dressing and tissue engineering. Polymers.

[8] Rivero S., Garcia M.A., Pinotti A. (2009). Composite and bi-layer films based on gelatin and chitosan. J. Food Eng..

[9] Pereda M., Ponce A., Marcovich N., Ruseckaite R., Martucci J. (2011). Chitosan-gelatin composites and bi-layer films with potential antimicrobial activity. Food Hydrocoll..

[10] Wang Y., Su L., Hou Y., Lin F., Xu C., Xue Y., Shi J., Wang X. (2022). A Biomimetic Composite Bilayer Dressing Composed of Alginate and Fibroin for Enhancing Full-Thickness Wound Healing. Macromol. Biosci..

[11] Zhang M., Wang G., Wang D., Zheng Y., Li Y., Meng W., Zhang X., Du F., Lee S. (2021). Ag@ MOF-loaded chitosan nanoparticle and polyvinyl alcohol/sodium alginate/chitosan bilayer dressing for wound healing applications. Int. J. Biol. Macromol..

[12] Olechno K., Basa A., Winnicka K. (2021). “Success depends on your backbone”—About the use of polymers as essential materials forming orodispersible films. Materials.

[13] De Luca I., Pedram P., Moeini A., Cerruti P., Peluso G., Di Salle A., Germann N. (2021). Nanotechnology development for formulating essential oils in wound dressing materials to promote the wound-healing process: A review. Appl. Sci..

[14] Pérez-Recalde M., Arias I.E.R., Hermida É.B. (2018). Could essential oils enhance biopolymers performance for wound healing? A systematic review. Phytomedicine.

[15] Osaili T.M., Dhanasekaran D.K., Zeb F., Faris M.E., Naja F., Radwan H., Ismail L.C., Hasan H., Hashim M., Obaid R.S. (2023). A status review on health-promoting properties and global regulation of essential oils. Molecules.

[16] Sternberg S.A., Wolfson C., Baumgarten M. (2000). Undetected dementia in community-dwelling older people: The Canadian study of health and aging. J. Am. Geriatr. Soc..

[17] Li A., Khan I.N., Khan I.U., Yousaf A.M., Shahzad Y. (2021). Gellan gum-based bilayer mucoadhesive films loaded with moxifloxacin hydrochloride and clove oil for possible treatment of periodontitis. Drug Des. Dev. Ther..

[18] Naik S., Raikar P., Ahmed M.G. (2019). Formulation and evaluation of chitosan films containing sparfloxacin for the treatment of periodontitis. J. Drug Deliv. Technol.

[19] Kanmani P., Rhim J.-W. (2014). Properties and characterization of bionanocomposite films prepared with various biopolymers and ZnO nanoparticles. Carbohydr. Polym..

[20] Zayed G.M., Abd-El Rasoul S., Ibrahim M.A., Saddik M.S., Alshora D.H. (2020). In vitro and in vivo characterization of domperidone-loaded fast dissolving buccal films. Saudi Pharm. J..

[21] Al-Mogherah A., Abbas M., Abdelazeem M. (2020). Optimization and evaluation of venlafaxine hydrochloride fast dissolving oral films. Saudi Pharm. J..

[22] Postolović K., Ljujić B., Kovačević M.M., Đorđević S., Nikolić S., Živanović S., Stanić Z. (2022). Optimization, characterization, and evaluation of carrageenan/alginate/poloxamer/curcumin hydrogel film as a functional wound dressing material. Mater. Today Commun..

[23] Wang J., Li Y., Gao Y., Xie Z., Zhou M., He Y., Wu H., Zhou W., Dong X., Yang Z. (2018). Cinnamon oil-loaded composite emulsion hydrogels with antibacterial activity prepared using concentrated emulsion templates. Ind. Crops Prod..

[24] Ebrahimzadeh S., Bari M.R., Hamishehkar H., Kafil H.S., Lim L.-T. (2021). Essential oils-loaded electrospun chitosan-poly (vinyl alcohol) nonwovens laminated on chitosan film as bilayer bioactive edible films. LWT.

[25] Razzaq F.A., Khalid S.H., Khan I.U., Asghar S. (2025). Multifunctional moxifloxacin and essential oil loaded sodium alginate/thiolated karaya gum hydrogel dressings for improved wound healing. Int. J. Biol. Macromol..

[26] Nesrinne S., Djamel A. (2017). Synthesis, characterization and rheological behavior of pH sensitive poly (acrylamide-co-acrylic acid) hydrogels. Arab. J. Chem..

[27] Anjum S., Arora A., Alam M., Gupta B. (2016). Development of antimicrobial and scar preventive chitosan hydrogel wound dressings. Int. J. Pharm..

[28] Vashisth P., Raghuwanshi N., Srivastava A.K., Singh H., Nagar H., Pruthi V. (2017). Ofloxacin loaded gellan/PVA nanofibers-Synthesis, characterization and evaluation of their gastroretentive/mucoadhesive drug delivery potential. Mater. Sci. Eng. C.

[29] Sanyang M., Sapuan S., Jawaid M., Ishak M., Sahari J. (2016). Development and characterization of sugar palm starch and poly (lactic acid) bilayer films. Carbohydr. Polym..

[30] Nilsuwan K., Benjakul S., Prodpran T. (2017). Properties, microstructure and heat seal ability of bilayer films based on fish gelatin and emulsified gelatin films. Food Biophys..

[31] Oustadi F., Haghbin Nazarpak M., Mansouri M., Ketabat F. (2022). Preparation, characterization, and drug release study of ibuprofen-loaded poly (vinyl alcohol)/poly (vinyl pyrrolidone) bilayer antibacterial membrane. Int. J. Polym. Mater. Polym. Biomater..

[32] Ahmady A.R., Razmjooee K., Saber-Samandari S., Toghraie D. (2022). Fabrication of chitosan-gelatin films incorporated with thymol-loaded alginate microparticles for controlled drug delivery, antibacterial activity and wound healing: In-vitro and in-vivo studies. Int. J. Biol. Macromol..

[33] Jafari A., Amirsadeghi A., Hassanajili S., Azarpira N. (2020). Bioactive antibacterial bilayer PCL/gelatin nanofibrous scaffold promotes full-thickness wound healing. Int. J. Pharm..

[34] Özay Y., Güzel S., Yumrutaş Ö., Pehlivanoğlu B., Erdoğdu İ.H., Yildirim Z., Türk B.A., Darcan S. (2019). Wound healing effect of kaempferol in diabetic and nondiabetic rats. J. Surg. Res..

[35] Abdel-Rashid R.S., El-Leithy E.S., Abdel-Monem R. (2021). Formulation and Evaluation of Topical Biodegradable Films Loaded with Levofloxacin Lipid Nanocarriers. AAPS PharmSciTech.

[36] Rezvanian M., Amin M.C.I.M., Ng S.-F. (2016). Development and physicochemical characterization of alginate composite film loaded with simvastatin as a potential wound dressing. Carbohydr. Polym..

[37] Junmahasathien T., Panraksa P., Protiarn P., Hormdee D., Noisombut R., Kantrong N., Jantrawut P. (2018). Preparation and evaluation of metronidazole-loaded pectin films for potentially targeting a microbial infection associated with periodontal disease. Polymers.

[38] Kittipongpatana O.S., Trisopon K., Wattanaarsakit P., Kittipongpatana N. (2022). Fabrication and characterization of orodispersible composite film from hydroxypropylmethyl cellulose-crosslinked carboxymethyl rice starch. Membranes.

[39] Preis M., Woertz C., Schneider K., Kukawka J., Broscheit J., Roewer N., Breitkreutz J. (2014). Design and evaluation of bilayered buccal film preparations for local administration of lidocaine hydrochloride. Eur. J. Pharm. Biopharm..

[40] Fernandes F.P., Fortes A.C., da Cruz Fonseca S.G., Breitkreutz J., Ferraz H.G. (2018). Manufacture and characterization of mucoadhesive buccal films based on pectin and gellan gum containing triamcinolone acetonide. Int. J. Polym. Sci..

[41] Khan B.A., Ullah S., Khan M.K., Uzair B., Menaa F., Braga V.A. (2020). Fabrication, physical characterizations, and in vitro, in vivo evaluation of ginger extract-loaded gelatin/poly (vinyl alcohol) hydrogel films against burn wound healing in animal model. AAPS PharmSciTech.

[42] Al-Harrasi A., Bhatia S., Al-Azri M.S., Ullah S., Najmi A., Albratty M., Meraya A.M., Mohan S., Aldawsari M.F. (2022). Effect of drying temperature on physical, chemical, and antioxidant properties of ginger oil loaded gelatin-sodium alginate edible films. Membranes.

[43] Rahim K., Saleha S., Zhu X., Huo L., Basit A., Franco O.L. (2017). Bacterial contribution in chronicity of wounds. Microb. Ecol..

[44] Kirketerp-Møller K., Jensen P.Ø., Fazli M., Madsen K.G., Pedersen J., Moser C., Tolker-Nielsen T., Høiby N., Givskov M., Bjarnsholt T. (2008). Distribution, organization, and ecology of bacteria in chronic wounds. J. Clin. Microbiol..

[45] Sánchez-González L., Cháfer M., Hernández M., Chiralt A., González-Martínez C. (2011). Antimicrobial activity of polysaccharide films containing essential oils. Food Control.

[46] Marotta S.M., Giarratana F., Parco A., Neri D., Ziino G., Giuffrida A., Panebianco A. (2016). Evaluation of the antibacterial activity of bergamot essential oils on different Listeria monocytogenes strains. Ital. J. Food Saf..

[47] Liakos I., Rizzello L., Scurr D.J., Pompa P.P., Bayer I.S., Athanassiou A. (2014). All-natural composite wound dressing films of essential oils encapsulated in sodium alginate with antimicrobial properties. Int. J. Pharm..

[48] Almutairi M.B.F., Alrouji M., Almuhanna Y., Asad M., Joseph B. (2022). In-vitro and in-vivo antibacterial effects of Frankincense oil and its interaction with some antibiotics against multidrug-resistant pathogens. Antibiotics.

[49] Blejan E.I., Popa D.E., Costea T., Cioacă A., Olariu L., Ghica M., Georgescu M., Stancov G., Arsene A.L. (2021). The in vitro antimicrobial activity of some essential oils from aromatic plants. Farmacia.

[50] Burt S. (2004). Essential oils: Their antibacterial properties and potential applications in foods—A review. Int. J. Food Microbiol..

[51] Ali B., Blunden G. (2003). Pharmacological and toxicological properties of Nigella sativa. Phytother. Res. Int. J. Devoted Pharmacol. Toxicol. Eval. Nat. Prod. Deriv..

[52] Sahiner M., Sagbas S., Bitlisli B.O. (2015). p (AAm/TA)-based IPN hydrogel films with antimicrobial and antioxidant properties for biomedical applications. J. Appl. Polym. Sci..

[53] Gulcin İ., Alwasel S.H. (2023). DPPH radical scavenging assay. Processes.

[54] Emami M., Zomorodian K., Yazdanpanah S., Ghasemi Y., Mirzaei E., Derakhshan M.A. (2024). Multifunctional Bi-layer collagen nanofiber-collagen/PLLA/Zataria multiflora essential oil nanofiber for wound healing: Antibacterial, antifungal and antioxidant properties. J. Drug Deliv. Sci. Technol..

[55] de Oliveira Fulgêncio G., Viana F.A.B., Silva R.O.S., Lobato F.C.F., Ribeiro R.R., Fanca J.R., Byrro R.M.D., Faraco A.A.G., da Silva Cunha-Júnior A. (2014). Mucoadhesive chitosan films as a potential ocular delivery system for ofloxacin: Preliminary in vitro studies. Vet. Ophthalmol..

[56] Agubata C.O., Okereke C., Nzekwe I.T., Onoja R.I., Obitte N.C. (2016). Development and evaluation of wound healing hydrogels based on a quinolone, hydroxypropyl methylcellulose and biodegradable microfibres. Eur. J. Pharm. Sci..

[57] Xiao Q., Gu X., Tan S. (2014). Drying process of sodium alginate films studied by two-dimensional correlation ATR-FTIR spectroscopy. Food Chem..

[58] Sahoo S., Chakraborti C.K., Behera P.K. (2012). Spectroscopic investigations of a ciprofloxacin/hpmc mucoadhesive suspension. Int. J. Appl. Pharm..

[59] Tang L., Hong B., Li T., Huang B. (2019). Development of bilayer films based on shellac and esterified cellulose nanocrystals for buccal drug delivery. Cellulose.

[60] Martins V.D., Cerqueira M.A., Fuciños P., Garrido-Maestu A., Curto J.M., Pastrana L.M. (2018). Active bi-layer cellulose-based films: Development and characterization. Cellulose.

[61] Naglah A.M., Al-Omar M.A., Almehizia A.A., AlKahtani H.M., Bhat M.A., Al-Shakliah N.S., Belgacem K., Majrashi B.M., Refat M.S., Adam A.M.A. (2021). Synthesis, thermogravimetric, and spectroscopic characterizations of three palladium metal (46) ofloxacin drug and amino acids mixed ligand complexes as advanced antimicrobial materials. J. Mol. Struct..

[62] Han Y., Yu M., Wang L. (2018). Physical and antimicrobial properties of sodium alginate/carboxymethyl cellulose films incorporated with cinnamon essential oil. Food Packag. Shelf Life.

[63] Zhang B., Liu Y., Wang H., Liu W., Cheong K.-l., Teng B. (2021). Characterization of seaweed polysaccharide-based bilayer films containing essential oils with antibacterial activity. LWT.

[64] Chalitangkoon J., Wongkittisin M., Monvisade P. (2020). Silver loaded hydroxyethylacryl chitosan/sodium alginate hydrogel films for controlled drug release wound dressings. Int. J. Biol. Macromol..

[65] Taşkın Çakıcı G. (2023). Nano TiO_2_-doped sodium alginate/hydroxypropyl methylcellulose synthesis of bionanocomposite membrane and its use in controlled release of anti-cancer drug 5-fluorouracil. Polym. Bull..

[66] Prezotti F.G., Cury B.S.F., Evangelista R.C. (2014). Mucoadhesive beads of gellan gum/pectin intended to controlled delivery of drugs. Carbohydr. Polym..

[67] Rade P.P., Garnaik B. (2020). Ofloxacin-loaded PLLA nanofibrous mats for wound dressing applications. ACS Appl. Bio Mater..

[68] Lindert S., Breitkreutz J. (2017). Oromucosal multilayer films for tailor-made, controlled drug delivery. Expert Opin. Drug Deliv..

[69] Sultan M., Elsayed H., Abdelhakim A.E.F., Taha G. (2022). Active packaging gelatin films based on chitosan/Arabic gum/coconut oil Pickering nano emulsions. J. Appl. Polym. Sci..

[70] Adrover A., Varani G., Paolicelli P., Petralito S., Di Muzio L., Casadei M.A., Tho I. (2018). Experimental and modeling study of drug release from HPMC-based erodible oral thin films. Pharmaceutics.

[71] Agwa M.M., Sabra S., Atwa N.A., Dahdooh H.A., Lithy R.M., Elmotasem H. (2022). Potential of frankincense essential oil-loaded whey protein nanoparticles embedded in frankincense resin as a wound healing film based on green technology. J. Drug Deliv. Sci. Technol..

[72] Pilicheva B., Uzunova Y., Bodurov I., Viraneva A., Exner G., Sotirov S., Yovcheva T., Marudova M. (2020). Layer-by-layer self-assembly films for buccal drug delivery: The effect of polymer cross-linking. J. Drug Deliv. Sci. Technol..

[73] Youssef A., Dudhipala N., Majumdar S. (2020). Ciprofloxacin loaded nanostructured lipid carriers incorporated into in-situ gels to improve management of bacterial endophthalmitis. Pharmaceutics.

[74] Alhallak M., Karpukhina N., Patel M. (2023). Triamcinolone acetonide release modelling from novel bilayer mucoadhesive films: An in vitro study. Dent. Mater..

[75] Sethi V., Kaur M., Thakur A., Rishi P., Kaushik A. (2022). Unravelling the role of hemp straw derived cellulose in CMC/PVA hydrogel for sustained release of fluoroquinolone antibiotic. Int. J. Biol. Macromol..

[76] Dharmalingam K., Anandalakshmi R. (2019). Fabrication, characterization and drug loading efficiency of citric acid crosslinked NaCMC-HPMC hydrogel films for wound healing drug delivery applications. Int. J. Biol. Macromol..

[77] Muta T., Parikh A., Kathawala K., Haidari H., Song Y., Thomas J., Garg S. (2020). Quality-by-design approach for the development of nano-sized tea tree oil formulation-impregnated biocompatible gel with antimicrobial properties. Pharmaceutics.

[78] Retnaningtyas E., Susatia B., Khotimah H., Rudijanto A., Abousouh A.A.A., Setiawan A. (2024). Centella asiatica transfersomes and Bergamot essential oil nanoemulsion combined in gel exhibited anti-photoaging effects on UVB-radiated BALB/c mice. J. King Saud Univ.-Sci..

[79] Agyare C., Osafo N., Boakye Y.D. (2018). Biomarkers of wound healing. Wound Healing-Current Perspectives.

[80] Fatima M., Iqbal Y., Ahmad M.M., Chatha S.A.S., Khan I.U., Hussain A.I. (2024). Fabrication and evaluation of CMC-Ag and CMC-Zn-based composite films as biobased wound dressings. Carbohydr. Polym. Technol. Appl..

[81] Saleem M., Asif M., Parveen A., Yaseen H.S., Saadullah M., Bashir A., Asif J., Arif M., Khan I.U., Khan R.U. (2021). Investigation of in vivo anti-inflammatory and anti-angiogenic attributes of coumarin-rich ethanolic extract of Melilotus indicus. Inflammopharmacology.

[82] Sethi J., Hotamisligil G. (2021). Metabolic messengers: Tumour necrosis factor. Nat. Metab..

[83] Rashid N., Khalid S.H., Ullah Khan I., Chauhdary Z., Mahmood H., Saleem A., Umair M., Asghar S. (2023). Curcumin-Loaded Bioactive Polymer Composite Film of PVA/Gelatin/Tannic Acid Downregulates the Pro-inflammatory Cytokines to Expedite Healing of Full-Thickness Wounds. ACS Omega.

[84] Arooj B., Asghar S., Saleem M., Khalid S.H., Asif M. (2023). Anti-inflammatory mechanisms of eucalyptol rich Eucalyptus globulus essential oil alone and in combination with flurbiprofen. Inflammopharmacology.

[85] Goswami A.G., Basu S., Huda F., Pant J., Ghosh Kar A., Banerjee T., Shukla V.K. (2022). An appraisal of vascular endothelial growth factor (VEGF): The dynamic molecule of wound healing and its current clinical applications. Growth Factors.

[86] Jun J.-I., Lau L.F. (2010). Cellular senescence controls fibrosis in wound healing. Aging.

[87] Kang B., Jia Z., Dong Y., Li W., Zhang W. (2024). Recombinant human annexin A5 accelerates diabetic wounds healing by regulating skin inflammation. Regen. Ther..

[88] Balkrishna A., Nain P., Chauhan A., Sharma N., Gupta A., Ranjan R., Varshney A. (2020). Super critical fluid extracted fatty acids from Withania somnifera seeds repair psoriasis-like skin lesions and attenuate pro-inflammatory cytokines (TNF-α and IL-6) release. Biomolecules.

[89] Dwivedi D., Dwivedi M., Malviya S., Singh V. (2017). Evaluation of wound healing, anti-microbial and antioxidant potential of Pongamia pinnata in wistar rats. J. Tradit. Complement. Med..

[90] Honnegowda T.M., Kumar P., Udupa E.G.P., Kumar S., Kumar U., Rao P. (2015). Role of angiogenesis and angiogenic factors in acute and chronic wound healing. Plast. Aesthetic Res..

[91] Bouter A., Carmeille R., Gounou C., Bouvet F., Degrelle S., Evain-Brion D., Brisson A. (2015). Annexin-A5 and cell membrane repair. Placenta.

[92] Coşkun G., Karaca E., Ozyurtlu M., Özbek S., Yermezler A., Çavuşoğlu İ. (2014). Histological evaluation of wound healing performance of electrospun poly (vinyl alcohol)/sodium alginate as wound dressing in vivo. Bio-Med. Mater. Eng..

[93] Mahmood H., Asif M., Khalid S.H., Khan I.U., Chauhdary Z., Razzaq F.A., Asghar S. (2023). Design of a multifunctional carrageenan-tannic acid wound dressing co-loaded with simvastatin and geranium oil. J. Drug Deliv. Sci. Technol..

[94] Iqbal Y., Chatha S.A.S., Chauhdary Z., Ijaz Hussain A., Khan I.U. (2023). Design and evaluation of moringa gum-based hydrogel dressings for cutaneous wound healing. J. Bioact. Compat. Polym..

[95] Čoma M., Fröhlichová L., Urban L., Zajíček R., Urban T., Szabo P., Novák Š., Fetissov V., Dvořánková B., Smetana K. (2021). Molecular changes underlying hypertrophic scarring following burns involve specific deregulations at all wound healing stages (inflammation, proliferation and maturation). Int. J. Mol. Sci..

