# Supplementary material for: Multifunctional Bilayer Wound Dressing Composed of Immediate Release Layer of Ofloxacin and Sustained Release Layer of Bergamot Oil
Source: Pharmaceutics. 2025 Dec 10;17(12):1589. doi: 10.3390/pharmaceutics17121589 (PMC12736944; doi:10.3390/pharmaceutics17121589)
Supplement: Supplementary file 1 [file pharmaceutics-17-01589-s001.zip › pharmaceutics-3900505-supplementary.pdf]

Supplementary file

Article

Multifunctional Bilayer Wound Dressing Composed of Immediate Release Layer of Ofloxacin and Sustained Release Layer of Bergamot Oil

Mehar Un Nisa [1], Ikram Ullah Khan [1,*], Yousaf Kamal [2], Usra [3], Zunera Chauhdary [4], Ghulam Hussain [3], Muhammad Irfan [1], Syed Haroon Khalid [1,5], Sajid Asghar [1], Hafeez Ullah Khan [6], Safirah Maheen [6], Syed Adnan Ali Shah [7,8] Abdulrahman Alshammari[9] and Thierry F. Vandamme [10]

[1]Department of Pharmaceutics, Faculty of Pharmaceutical Sciences, Government College University Faisalabad, Faisalabad 38000, Pakistan; mehar.2172@gmail.com (M.U.N) Ikramglt@gmail.com (I.U.K), manipharma1@gmail.com (M.I.); sajuhappa@gmail.com (S.A.); haroonkhalid80@gmail.com

[2]Hamdard Institute of Pharmaceutical Sciences, Hamdard University Karachi, Islamabad Campus, Islamabad 45550, Pakistan; yousafpharmacist1@gmail.com

[3]Department of Physiology, Faculty of Life Sciences, Government College University, Faisalabad 38000, Pakistan; usrakhan1990@gmail.com (U), hussain806@gmail.com (G.H.)

[4]Department of Pharmacology, Faculty of Pharmaceutical Sciences, Government College University Faisalabad, Faisalabad 38000, Pakistan; zunerach@yahoo.com

[5]Department of Pharmaceutics, Faculty of Pharmacy, Universiti Teknologi Mara, Puncak Alam, 42300, Selangor, Malaysia

[6]Department of Pharmaceutics, College of Pharmacy, University of Sargodha, Sargodha, 40100, Pakistan. hafeezullah.khan@uos.edu.pk, msafirah@yahoo.com

[7]Faculty of Pharmacy, Universiti Teknologi MARA Cawangan Selangor Kampus Puncak Alam, Bandar Puncak Alam 42300, Selangor D. E., Malaysia; syedadnan@uitm.edu.my

[8]Atta-ur-Rahman Institute for Natural Product Discovery (AuRIns), Universiti Teknologi MARA Cawangan Selangor Kampus Puncak Alam, Bandar Puncak Alam42300, Selangor D. E., Malaysia; syedadnan@uitm.edu.my

[9]Department of Pharmacology and Toxicology, College of Pharmacy, King Saud University, P.O. Box 2455, Riyadh 11451, Saudi Arabia; abdalshammari@ksu.edu.sa (A.A.)

[10]Centre de Recherche en Biomédecine de Strasbourg (CRBS), Inserm/Unistra, UMR 1260 Regenerative NanoMedecine, Université de Strasbourg, 1 Rue Eugène Boeckel, 67000 Strasbourg, France; vandamme@unistra.fr

* **Correspondence:** author: Ikram Ullah Khan, email: ikramglt@gmail.com

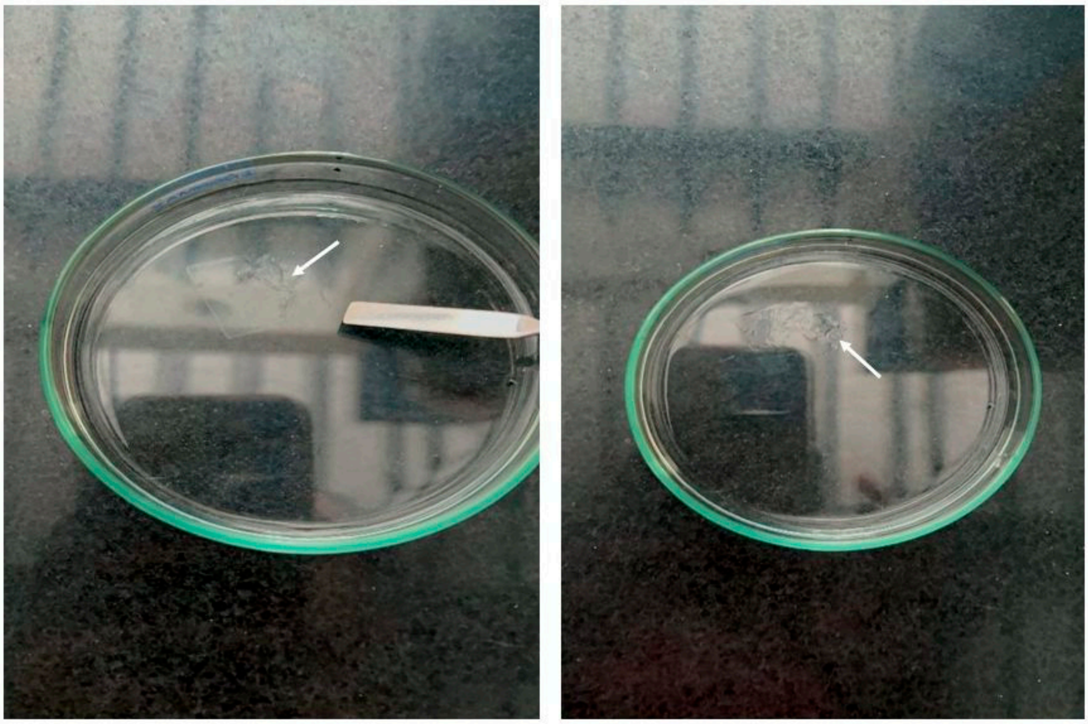

Figure S1. Optical image of disintegration of D2.

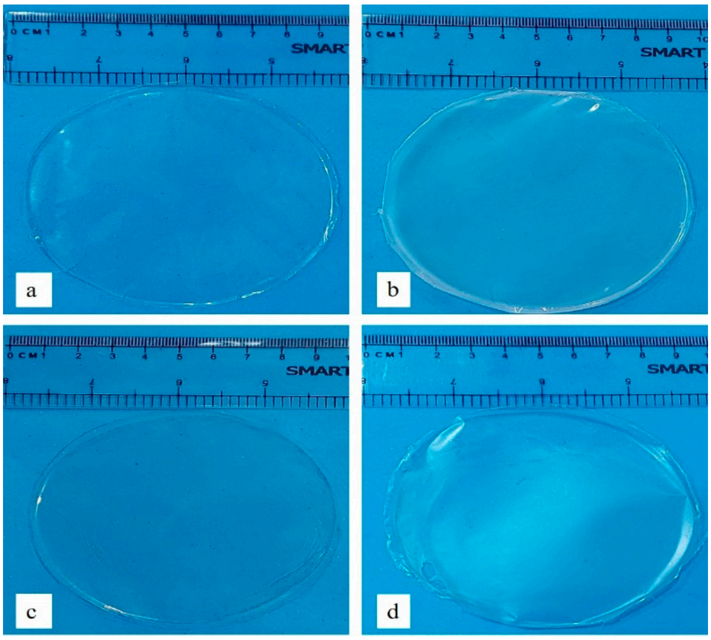

Figure S2. Physical appearance of prepared films (a) blank film (SA blank), (b) oil-loaded film (B2), (c) blank HPMC and HEC film (D blank), and (d) drug-loaded film (D2).

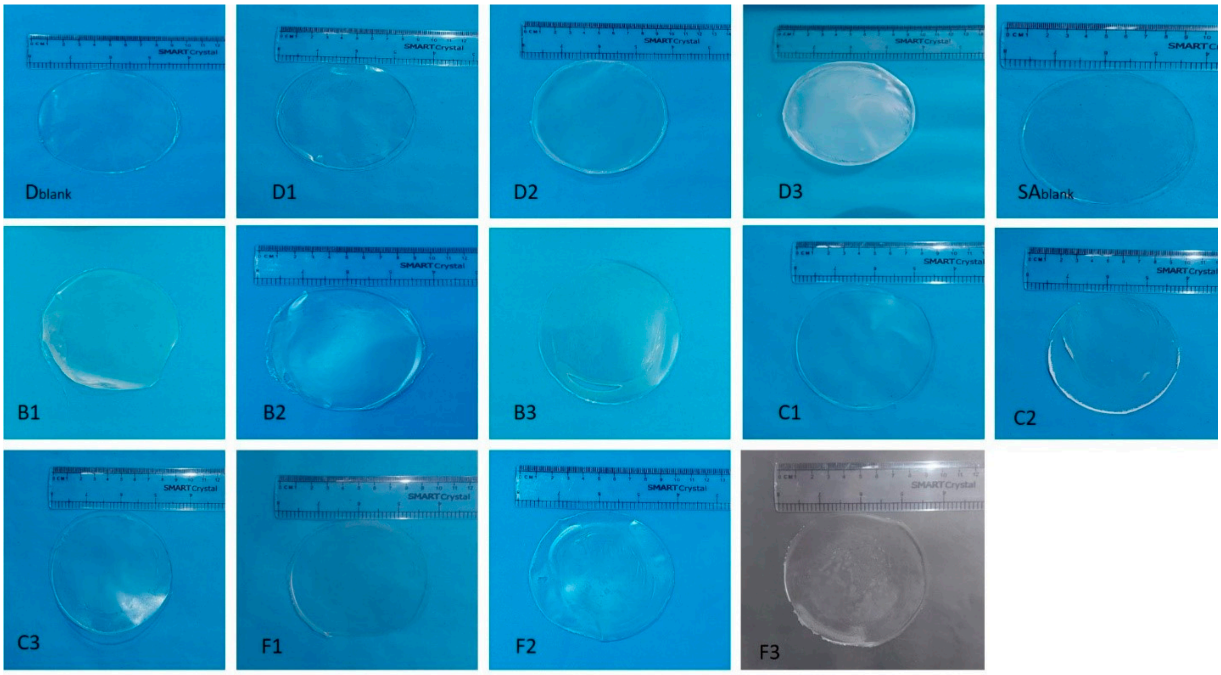

Figure S3. Optical images of single-layer films.

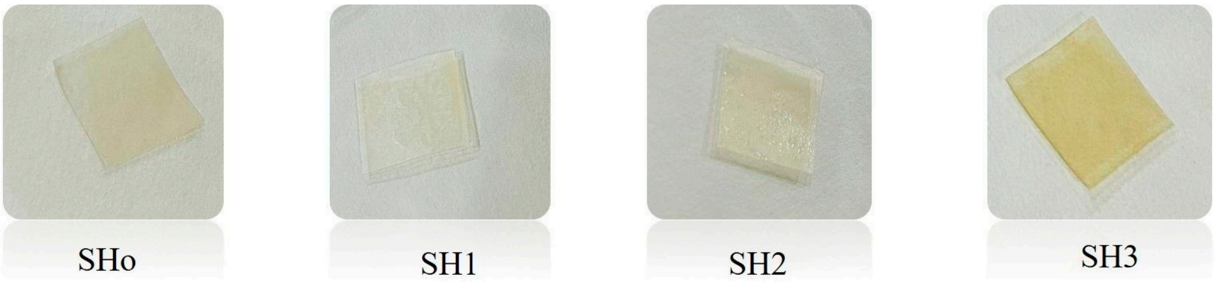

Figure S4. Optical images of 1.5 x 1.5 cm^2 patches of bilayer formulation.

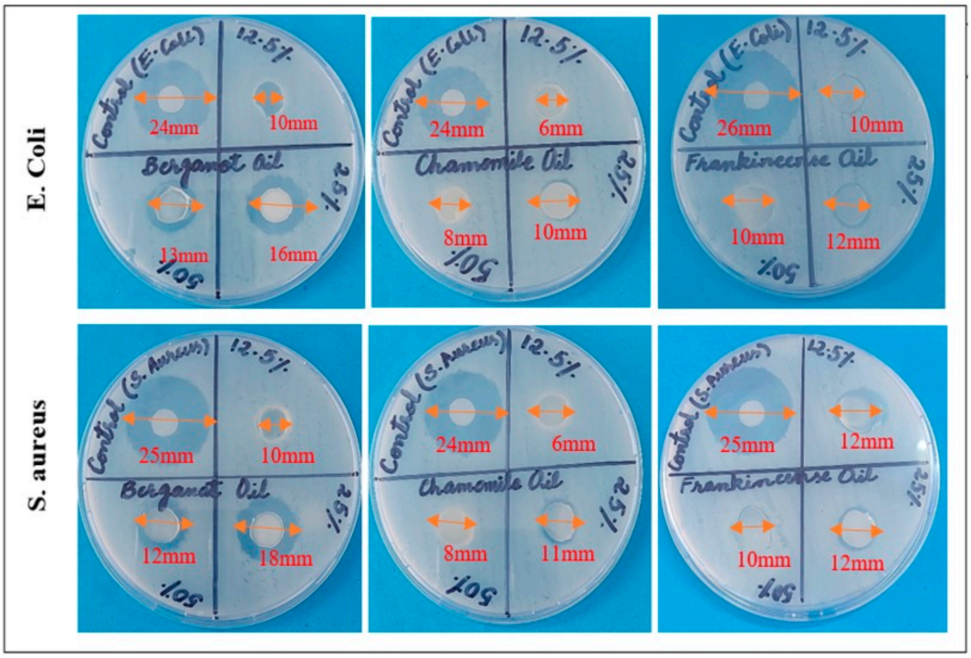

Figure S5. Comparison of antibacterial activity of film loaded with oils at different concentrations against *S. aureus* and *E. coli.*

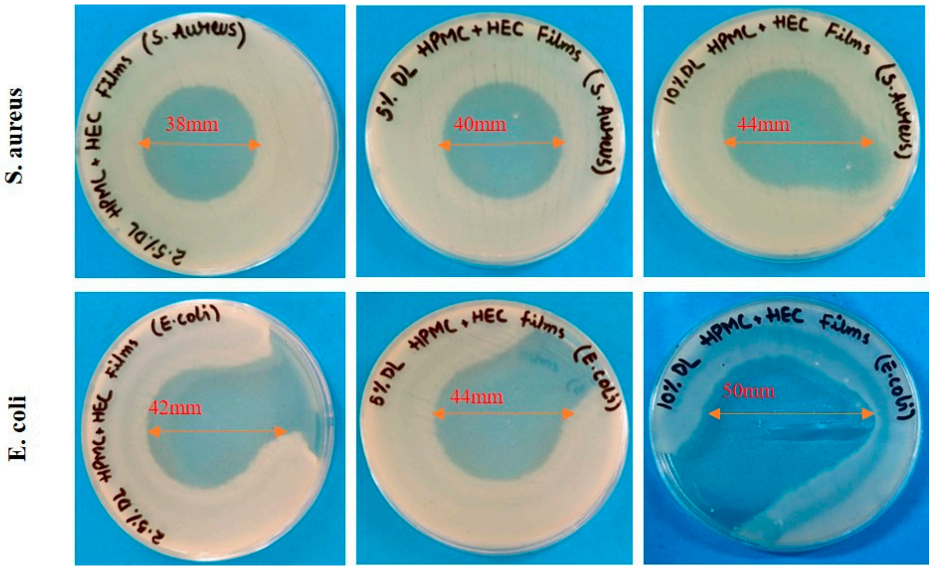

Figure S6. Comparison of anti-bacterial activity films loaded with drug at different concentrations against *S. aureus* and *E. coli.*
